# Specific carotenoid pigments in the diet and a bit of oxidative stress in the recipe for producing red carotenoid-based signals

**DOI:** 10.7717/peerj.2237

**Published:** 2016-09-01

**Authors:** Esther García-de Blas, Rafael Mateo, Carlos Alonso-Alvarez

**Affiliations:** 1 Instituto de Investigación en Recursos Cinegéticos (IREC), CSIC-UCLM-JCCM, Ciudad Real, Spain; 2 Ecología Evolituva, Museo Nacional de Ciencias Naturales (MNCN), Consejo Superior de Investigaciones Científicas (CSIC), Madrid, Spain

**Keywords:** Sexual signaling, Sexual selection, Carotenoids, Color signaling, Oxidative stress, Avian coloration, Carotenoid supplementation, Handicap theory, Carotenoid transformation, Hormesis

## Abstract

Colorful ornaments have been the focus of sexual selection studies since the work of Darwin. Yellow to red coloration is often produced by carotenoid pigments. Different hypotheses have been formulated to explain the evolution of these traits as signals of individual quality. Many of these hypotheses involve the existence of a signal production cost. The carotenoids necessary for signaling can only be obtained from food. In this line, carotenoid-based signals could reveal an individual’s capacity to find sufficient dietary pigments. However, the ingested carotenoids are often yellow and became transformed by the organism to produce pigments of more intense color (red ketocarotenoids). Biotransformation should involve oxidation reactions, although the exact mechanism is poorly known. We tested the hypothesis that carotenoid biotransformation could be costly because a certain level of oxidative stress is required to correctly perform the conversion. The carotenoid-based signals could thus reveal the efficiency of the owner in successfully managing this challenge. In a bird with ketocarotenoid-based ornaments (the red-legged partridge; *Alectoris rufa*), the availability of different carotenoids in the diet (i.e. astaxanthin, zeaxanthin and lutein) and oxidative stress were manipulated. The carotenoid composition was analyzed and quantified in the ornaments, blood, liver and fat. A number of oxidative stress biomarkers were also measured in the same tissues. First, we found that color and pigment levels in the ornaments depended on food levels of those carotenoids used as substrates in biotransformation. Second, we found that birds exposed to mild levels of a free radical generator (diquat) developed redder bills and deposited higher amounts of ketocarotenoids (astaxanthin) in ornaments. Moreover, the same diquat-exposed birds also showed a weaker resistance to hemolysis when their erythrocytes were exposed to free radicals, with females also enduring higher oxidative damage in plasma lipids. Thus, higher color production would be linked to higher oxidative stress, supporting the biotransformation hypothesis. The recent discovery of an avian oxygenase enzyme involved in converting yellow to red carotenoids may support our results. Nonetheless, the effect could also depend on the abundance of specific substrate carotenoids in the diet. Birds fed with proportionally higher levels of zeaxanthin showed the reddest ornaments with the highest astaxanthin concentrations. Moreover, these birds tended to show the strongest diquat-mediated effect. Therefore, in the evolution of carotenoid-based sexual signals, a biotransformation cost derived from maintaining a well-adjusted redox machinery could coexist with a cost linked to carotenoid acquisition and allocation (i.e. a resource allocation trade-off).

## Introduction

Colored ornaments in animals have attracted the attention of evolutionary biologists since Charles Darwin, who suggested that most conspicuously colored traits are the product of sexual selection (Darwin, 1871). Colored ornaments should provide some advantage when competing for a mate with same sex individuals (intrasexual selection) or by being more attractive to the choosing sex (intersexual selection; Andersson, 1994). In many cases, colored traits inform competitors or potential mates about the quality of the owner. However, the trait should generate some benefit for both emitter and receptor to be considered as a signal (Hasson, 1997; Bradbury & Vehrenkamp, 1998; Maynard Smith & Harper, 2003). This can occur by the transmission of information in a reliable (non-falsifiable) way (Maynard Smith & Harper, 2003).

Zahavi (1975) proposed the “handicap principle,” in which the reliability of the signal is due to its production/maintenance costs. The expression of a signal would proportionally be more costly for low-quality individuals compared to high-quality ones (Grafen, 1990; also Getty, 2006), the former being unable to signal or signaling in an inefficient way.

Carotenoids are natural pigments with immune-stimulant and antioxidant properties (Britton, Liaaen-Jensen & Pfander, 2009) that are present in the integument of many vertebrate species, generating conspicuously colored traits (e.g. Brush, 1990; Stradi, 1998; McGraw, 2006). The most obvious cost of carotenoid-based signals is the increase of conspicuousness that would raise the risk of predation (e.g. Godin & McDonough, 2003). This idea was suggested as early as Darwin (1871), regarding colorful ornaments but without citing the pigments.

The second cost associated with these traits is related to the fact that carotenoids cannot be synthesized de novo by the organism, but are only obtained from food (Britton, Liaaen-Jensen & Pfander, 2009; McGraw, 2006). Assuming that carotenoids are relatively scarce in food, colored individuals should pay a cost in terms of energy or time spent searching for pigments, which was suggested by Endler (1980) and Endler (1983) in fish studies (also Kodric-Brown, 1985; see in birds Hill, 1990; McGraw, 2006). This hypothesis is difficult to test and has garnered mixed support, at least in avian species (reviewed in Hill (2006)), which is probably the taxon where carotenoid-based signaling has been studied most in-depth (McGraw, 2006; Pérez-Rodríguez, 2009; Simons, Cohen & Verhulst, 2012). Subsequently, Lozano (1994) was the first to emphasize the physiologically specific roles of carotenoids in an evolutionary context, suggesting that investing large amounts of pigment in signaling could compromise the immune system. This idea seems to be well supported at least for some inflammatory responses (phytohaemagglutinin skin test) in birds (reviewed in Simons, Cohen & Verhulst (2012)). Subsequently, von Schantz et al. (1999) followed a similar reasoning but regarding the antioxidant properties of the pigments, proposing that investing in coloration would challenge the individual’s capacity to combat oxidative stress. This type of stress is the result of an imbalance between the production of reactive oxygen and nitrogen species (RONS) by cell respiration and immune responses and the state (levels and efficiency) of the antioxidant defenses (Halliwell & Gutteridge, 2007). An evolutionary trade-off (van Noordwijk & de Jong, 1986) in the investment of the carotenoid resources between self-maintenance (antioxidant defense) and reproduction (sexual signaling) could thus be established (Møller et al., 2000; Alonso-Alvarez et al., 2008). The von Schantz et al. (1999) hypothesis has gained popularity (e.g. Blount et al., 2003; Alonso-Alvarez et al., 2004; Hörak et al., 2007), probably because it unifies the physiological components of trait expression, since the immune response is at least partially regulated by the oxidative machinery (Halliwell & Gutteridge, 2007; Sorci & Faivre, 2009; Vallverdú-Coll et al., 2015).

Nonetheless, the antioxidant role of those carotenoids involved in sexual signaling has been questioned. This criticism has mostly arisen from the weakness of some correlations between carotenoid blood levels and certain measures of antioxidant capacity or oxidative damage in avian species (Costantini & Møller, 2008; Isaksson & Andersson, 2008). However, a meta-analysis on the published literature of this taxon seems to support the carotenoid antioxidant function, although the results were not robust (Simons, Cohen & Verhulst, 2012).

Importantly, the carotenoid molecules giving color to the ornaments are frequently not the same as those carotenoids obtained from the diet and circulating in the blood (e.g. fishes: Hata & Hata (1972) and Ohkubo et al. (1999); birds: McGraw (2006) and references therein). This issue may be key to understanding the cost of the signal, but many obscure points are as yet not understood. In particular, the site (tissue) where carotenoids are transformed and the type of biochemical processes involved in such transformations are little understood.

In avian species, the liver was the first tissue proposed as a potential biotransformation site (Brush & Power, 1976; Brush, 1990) because it stores large amounts of carotenoids and it is the main ‘laboratory’ of the organism (Blem, 2000; Britton, Liaaen-Jensen & Pfander, 2009). Carotenoid biotransformation in the liver could compete with the activity of enzymes involved in detoxification (Blem, 2000; Hill & Johnson, 2012). Hence, the fact that this vital organ could be involved could affect our understanding of the costs derived from color production. Carotenoid transformation in the liver was supported by studies in crossbills (*Loxia curvirostra*), which found the pigment used for coloration in the liver and blood (del Val et al., 2009a; del Val et al., 2009b; see also Hill & Johnson, 2012). Studies in many other bird species, however, did not find this and instead suggested that the ornament is the main transforming site (McGraw, 2004; McGraw, 2009; García-de Blas et al., 2014), which would perhaps be less important for survival compared to the liver.

To understand how carotenoids are transformed we first need to know the biochemical route followed from substrate pigments to ornamental carotenoids, including the intermediate compounds (McGraw, 2006; Britton, Liaaen-Jensen & Pfander, 2009). Lutein and zeaxanthin are the most abundant carotenoids in the diet and blood of birds (McGraw, 2006). Red ornaments displayed by many animal species are often the result of biotransformation of the cited yellow hydroxycarotenoids in red ketocarotenoids such as astaxanthin or canthaxanthin (McGraw, 2006). The pathway followed from hydroxy- to ketocarotenoids requires hydrogenation and oxidation reactions. The existence in vertebrates of specific enzymes (hydroxylases and 4-oxygenases (i.e. ketolases)) was first proposed (McGraw, 2006; Hill & Johnson, 2012) and subsequently demonstrated in birds (see Lopes et al. (2016) and Mundy et al. (2016) describing a candidate oxygenase). In this regard, Hill & Johnson (2012) and Johnson & Hill (2013) have recently suggested that the oxidative status of the organism could influence the activity of these enzymes, with the carotenoid-based signals, in some way, revealing the individual’s capacity to efficiently manage oxidative stress. The basic content of this idea was earlier formulated by Völker (1957) when trying to explain why wild birds often lost their color in captivity. He proposed that this phenomenon is the result of impairment in the oxidative metabolism involved in carotenoid transformations. Although this could have deep implications for understanding the proximate costs of animal signaling, the hypothesis has not been experimentally tested until now.

In the present study, the red-legged partridge (*Alectoris rufa*) was used as the model species. This gallinacean shows red ornaments (bill, eye rings, and legs) mostly produced by astaxanthin and papilioerythrinone ketocarotenoids (García-de Blas et al., 2013; García-de Blas et al., 2014). We have experimentally shown that red head traits of males are used by females to adjust their reproductive investment, suggesting that these ornaments are indeed involved in sexual selection (Alonso-Alvarez et al., 2012). Experiments have also shown a relationship between integumentary coloration (and circulating carotenoid levels) and individual quality in terms of immune capacity (Pérez-Rodríguez & Viñuela, 2008; Perez-Rodriguez et al., 2008; Mougeot et al., 2009). Redder birds also show a better resistance to oxidative stress when exposed to an immune challenge (Pérez-Rodríguez, Mougeot & Alonso-Alvarez, 2010). Moreover, young partridges exposed to high oxidative stress produced paler red traits and circulated lower blood carotenoid levels in adulthood (Alonso-Alvarez & Galván, 2011). We have also described that astaxanthin and papilioerythrinone pigments are not present in blood, liver or fat, which indicates that pigment transformation takes place at the ornament site (García-de Blas et al., 2013; García-de Blas et al., 2014; García-de Blas, Mateo & Alonso-Alvarez, 2015). We have proposed that astaxanthin and papilioerythrinone should be derived from zeaxanthin and lutein in food, respectively (i.e. García-de Blas et al., 2014), on the basis of published biochemical pathways (McGraw, 2006; LaFountain, Frank & Prum, 2013). Lutein and zeaxanthin, in this order, are the most abundant carotenoids in the blood of this (García-de Blas et al., 2013) and many other bird species (McGraw, 2006). As previously noted, the biotransformation of these compounds should involve oxidative reactions (McGraw, 2006). Dietary lutein would be transformed to papilioerythrinone after one 4-oxidation and one dehydrogenation reactions, whereas dietary zeaxanthin would be converted into astaxanthin by two 4-oxidations (McGraw, 2006; LaFountain, Frank & Prum, 2013; García-de Blas et al., 2014).

Here, the carotenoid content of the diet of captive red-legged partridges was manipulated, subsequently exposing birds to an oxidative challenge. Our aims were (1) to reveal the metabolic pathway from dietary carotenoids to those deposited in the ornaments, (2) to verify the contribution to integument coloration of each dietary carotenoid, and (3) to determine if oxidative stress can influence color and the individual capacity to transform substrate carotenoids into those carotenoids allocated to ornaments. In this order, some birds received food supplemented with different zeaxanthin vs. lutein proportions, whereas other individuals received astaxanthin. In order to induce a higher oxidative stress, half of the birds in each treatment were also exposed to a free radical generator (diquat) in drinking water (Galvan & Alonso-Alvarez, 2009; see also Koch & Hill, in press). We first predicted that a higher proportion of zeaxanthin in the diet should increase astaxanthin levels in ornaments whereas a higher proportion of lutein should instead raise the papilioerythrinone concentration. Since astaxanthin is the most abundant pigment in ornaments (García-de Blas et al., 2013; García-de Blas et al., 2014), the group receiving dietary astaxanthin should a priori produce the reddest color and the highest astaxanthin concentrations in bare parts because no transformations would be required (Negro & Garrido-Fernández, 2000). If transformations depend on specific enzymes inducing oxidative reactions, we can first predict that the oxidative challenge (higher availability of free radicals) could inhibit them by impairing/destabilizing the enzyme such as in the case of well-known antioxidant enzymes whose activity is decreased by high oxidative stress (e.g. glutathione synthase; Halliwell & Gutteridge, 2007). This would lead to paler birds with lower ketocarotenoid levels in ornaments. Alternatively, if the oxidative challenge is mild, these reactions could be favored in a sort of compensatory (hormetic) response (e.g. Costantini, Metcalfe & Monaghan, 2010). This would lead to redder colors and higher ketocarotenoid levels in ornaments and specific transforming sites (i.e. liver; see above). We can only speculate on this mechanism as the nature of the ketolase enzymes is still poorly understood. Their activity should a priori depend on oxygen availability (Fraser, Miura & Misawa, 1997; Schoefs et al., 2001). Superoxide or hydrogen peroxide generated by diquat redox cycling (Fussell et al., 2011; Koch & Hill, in press) could perhaps provide this oxygen required for oxygenase activity and/or activate redox signaling pathways increasing enzyme transcription. In fact, superoxide and hydrogen peroxide can act as prime redox signaling molecules activating many different cell pathways (e.g. Hurd & Murphy, 2009). Nonetheless, free radicals derived from diquat redox cycling could also directly promote oxidation of dietary pigments. This last possibility should, however, imply increased ketocarotenoid levels in any body site where pigments and diquat-derived molecules interact (the blood should be the first site after absorption).

## Material and Methods

### Manipulation of carotenoid content in food

In order to manipulate the carotenoid content of the diet, we collaborated with a company dedicated to producing animal pelleted feed (INALSA; Ciudad Real, Spain; http://www.piensos-inalsa.com/contenido/perdices.htm; INALSA-UCLM agreement signed on May 25, 2012). We preferred to manipulate carotenoid levels in food because carotenoids diluted in drinking water (1) can directly pigment head traits due to splashing (previous observations in this and other species) and (2) would have interfered with our oxidative stress manipulation. We supplied a free radical generator (diquat; see below) in water. Carotenoids and diquat in the same solution would have reacted producing pro-oxidant carotenoid metabolites (e.g. El-Agamey & McGarvey, 2008). Alternatively, the use of two different water dispensers for each type of treatment would not have guaranteed a similar consumption of each solution.

The manipulation of carotenoid levels in the pellets was made on a basal commercial diet normally used during reproduction of captive red-legged partridges, containing wheat, barley, corn and soy in different proportions (INALSA, Zaragoza, Spain). This feed did not contain any additional carotenoid to those naturally present in the grain (Panfili, Fratianni & Irano, 2004) and it was mixed with the different commercial carotenoids resulting in the final feed. Commercial pigments used to prepare the different diets for the experiment were CROMO ORO Classic (min. lutein 16 g/Kg and min. zeaxanthin 0.90 g/Kg), provided by DISPROQUIMA (Barcelona, Spain), OPTISHARP™ (Zeaxanthin 5% CWS/S-TG), provided by DSM Nutritional Products (Switzerland) and CAROPHYLL® Pink (Astaxanthin 10% CWS), provided by DSM Nutritional Products (Madrid, Spain). The adequate amounts of each pigment to add to the food were calculated taking into account the quantities of total carotenoids authorized for poultry feed (Directive 70/524/EEC, Communication 2004/C 50/01). Manipulation of carotenoid levels in the diet should resemble natural scenarios (Koch, Wilson & Hill, 2016). However, the natural carotenoid content in the diet of wild red-legged partridges is currently unknown. We should, nonetheless, consider that body carotenoid levels of wild partridges are significantly higher than levels in captive birds that usually receive carotenoid supplements (Table 9S in García-de Blas, Mateo & Alonso-Alvarez (2015)). This suggests that our supplements would not produce unnatural phenotypes. Moreover, no negative effect due to a hypothetical pharmacological level was detected in terms of survival, body mass, reproductive output (egg production) or oxidative stress levels (Results).

Pellets were elaborated following the habitual method of commercial feed preparation by using large-scale mills (Pietsch, 2005). This process yielded perfectly homogeneous pellets, similar in size and color to base feed, avoiding the pigmentation of the head of the birds by direct contact. Diet 1 (Control) was the basal diet. Diets 2 and 3 contained lutein and zeaxanthin in different proportions: Diet 2 (called LutZea) contained approximately 73% lutein and 27% zeaxanthin, and diet 3 (ZeaLut) was formed by 52% lutein and 48% zeaxanthin. Thus, diet 2 represented proportions often found in the natural diet of granivorous birds (McGraw, 2006), whereas diet 3 was a diet enriched for zeaxanthin. Diet 4 was supplemented with astaxanthin (Ast). Carotenoid, tocopherol and retinol content of each type of pellet are shown in Table 1. Unexpected differences in tocopherol and retinol levels among treatments were found. This was probably due to the protective antioxidant action of carotenoids on vitamins present in the basal feed during the pelleting process, which involves high pressures and temperatures (Pietsch, 2005), and to differences in the composition of supplements not detected during the formulation of each diet. Retinol and tocopherol are antioxidant vitamins involved in mutual recycling processes with carotenoids (Mortensen, Skibsted & Truscott, 2001; Catoni, Peters & Schaefer, 2008; Surai, 2012). To discard the influence of this potential bias, tocopherol and retinol levels in every analyzed tissue (ornaments, plasma, liver and fat) were quantified and included as covariates in all statistical models (below).

**Table 1:** Composition of a sample of each different type of food used in the experiment. For the names of the diets (see section Manipulation of carotenoid content in food).

Name diet	Lutein	Zeaxanthin	Astaxanthin	Total carotenoids	Retinol	Tocopherol
Control	1.33	0.69	0	1.96	2.5	8.3
LutZea	24.77	9.32	0	34.09	3.5	10.9
ZeaLut	17.64	18.6	0	36.24	10.4	15.1
Ast	5.3	4.8	22.87	32.97	14.4	18.5

### Experimental procedure

The study was carried out at the Dehesa de Galiana experimental facilities (Instituto de Investigación en Recursos Cinegéticos and Diputación Provincial, Ciudad Real, Spain). The protocol was approved by the University of Castilla-La Mancha’s Committee on Ethics and Animal Experimentation (approval number 1011.01). It was conducted on captive-born, one-year-old red-legged partridges provided by a governmental breeding facility (Chinchilla, Albacete, Spain). We used 182 adult partridges forming 91 pairs that were kept in outdoor cages (1 × 0.5 × 0.4 m, each pair) under natural photoperiods and temperatures. No birds died during the study, but ten birds were removed from the experiment (and statistical analyses) due to escapes during handling (treatment groups did not differ in these exclusions, all χ^2^, *P* > 0.12). In these cases, replacement birds were incorporated to keep pairs in similar conditions, but the new birds were not included in posterior samplings. The sex of individuals was determined genetically following Griffiths et al. (1998). Pairs were randomly divided into four groups that received one of the four diets. The sample size for Control, LutZea and ZeaLut groups was 23 pairs, and 22 pairs for the Ast group. Possible differences between groups in terms of food intake were checked during the experiment by weighing the pellet mass in feeders of a subsample of 10 pairs per group during one week, with no difference being detected (repeated-measures ANOVA; *F*_3,80_ = 0.732, *P* = 0.536). The experiment was carried out during the reproductive period (April–June), when the color expression of integuments is the greatest (Pérez-Rodríguez, 2008).

On April 11 (“day 0”), a blood sample and a color measurement (below) of each ornament (eye ring, bill, and legs) from each partridge were taken in order to determine pre-treatment color and blood levels of pigments and other physiological variables (below). Color measurements and blood samples were again taken on May 29 (“day 48;” intermediate sample). A third color and blood sampling was performed at the end of the experiment (July 2; “day 82”). One mL of blood was taken from the jugular vein, each time using heparinized syringes. Blood was centrifuged at 10,000 × g for 10 min at 4 °C to separate plasma from the cell fraction. Both were stored separately at −80 °C for later analysis. Before centrifugation, an aliquot of each blood sample was taken to calculate the hematocrit and resistance of erythrocytes against an oxidative challenge (see below).

On May 30, just after the second sampling, half of each treatment group (*n* = 45 pairs) were randomly allocated to the oxidative challenge. Of them, 11 pairs were from Control, ZeaLut, and Ast groups, and 12 pairs from the LutZea treatment. These birds were treated with diquat dibromide added to drinking water. The commercial product “Reglone” (Syngenta, Madrid) was used (20% w/v of diquat dibromide in water). Diquat dibromide is a redox cycler that is transformed to a free radical which, in reaction with molecular oxygen, produces superoxide and other redox products (e.g. Sewalk, Brewer & Hoffman, 2000; Zeman et al., 2005; Xu et al., 2007). The diquat bromide dose (i.e. 0.50 mL/L Reglone in drinking water; Reglone contains 20% w/v of diquat dibromide in water) was established on the basis of a pilot study and the results obtained in previous work in the same species, which reported no body mass changes but increased lipid oxidative damage in erythrocytes (see Supporting Fig. 1 in Alonso-Alvarez & Galván (2011); see also Galvan & Alonso-Alvarez, 2009).

### Color measurements

The coloration of eye-rings and bills of red-legged partridges was assessed by using a portable spectrophotometer (Minolta CM-2600D, Tokyo). Hue values were calculated by using the formula of Saks, Mcgraw & Hõrak (2003) for brightness (B) of different colors (i.e. hue = arctan {[(By−Bb)/BT]/[(Br−Bg)/BT]}, where yellow (y) is the addition of percentage reflectance within the 550–625 nm range, red (r) = 625–700 nm, blue (b) = 400–475 nm, green (g) = 475–550 nm and T is total brightness). BT obtained from our spectrophotometer (360–700 nm) was added as a covariate to models testing the hue (see Statistical Analyses), since the original formula includes BT in both numerator and denominator, thus canceling out its effect. Repeatabilities of triplicate spectrophotometric measurements were significant for both traits (*r* > 0.68, *P* < 0.001), with mean values for each sample being used.

Leg color was assessed by means of digital photographs (Nikon D-3100; see also García-de Blas et al., 2013) because the probe of our spectrophotometer did not adapt well to the leg surface (also Alonso-Alvarez & Galván, 2011). In this case, the birds were placed in the same position under standardized indoor light conditions (Kaiser Repro Lighting Unit; Repro Base with lights RB260 2 × 11 W 6,000 °K; Kaiser Fototechnik, Buchen) with the camera (Nikon D-3100) always set to the same focus and conditions. A red color chip (Kodak, NY, USA) was placed close to the legs in order to control for subtle changes in environmental light, adding the hue values of the chip as a covariate to models testing leg color (*Statistical analyses*). Pictures were analyzed by a technician blinded to the birds’ identity. The color intensity of the central area of one of the tarsi was determined in adults by recording mean red, green and blue values (RGB system; e.g. Alonso-Alvarez et al., 2008) using Adobe Photoshop CS3. Hue was determined after conversion of RGB values by using the Foley & Van Dam (1984) algorithm. Repeatability of picture measurements taken twice from a different sample of red-legged partridges was high (*r* > 0.90, *P* < 0.001, *n* = 71; Alonso-Alvarez & Galván, 2011). Since lower hue values obtained from spectrophotometer measures or pictures indicated higher redness, the sign of the hue variables was reversed (multiplied by −1) to simplify interpretations. The term “redness” was thus used to describe the hue inverse.

### Quantification of carotenoids and vitamins

The analyses of carotenoids, and vitamins A and E in internal tissues (i.e. plasma, liver, and subcutaneous fat) and colored integuments were performed by HPLC-DAD-FLD following the methods described by Rodríguez-Estival et al. (2010); García-de Blas et al. (2011) and García-de Blas et al. (2013). Carotenoid levels are total values adding the levels of esterified and free forms for each specific pigment. Standards of lutein, zeaxanthin, canthaxanthin, astaxanthin, astaxanthin monopalmitate and astaxanthin dipalmitate were purchased from CaroteNature (Lupsingen, Switzerland). Retinyl acetate (used as an internal standard) and standards of retinol and α-tocopherol were provided by Sigma-Aldrich. Carotenoid and vitamin concentrations were expressed as nmoles per gram of tissue.

### Resistance to hemolysis under free radical exposure

The resistance of red blood cells to hemolysis under exposure to a free radical generator was assessed. Whole blood was exposed to a thermo-controlled free radical aggression by adding 2,2-azobis-(aminodinopropane) hydrochloride (AAPH) (Rojas Wahl et al., 1998). Previous work has shown that if at least one component of the antiradical detoxification system is impaired, the hemolysis curve shows a shift towards shorter times (Blache & Prost, 1992; Girard et al., 2005). This test, therefore, provides an assessment of resistance to oxidative stress because all families of free radical scavengers present in the blood are mobilized to fight off the oxidant attack (e.g. Blache & Prost, 1992; Lesgards et al., 2002; Girard et al., 2005). Ten microliters of the blood of adult birds were immediately diluted and mixed with 365 μL of KRL buffer (for 50 mL: 0.020 g of KHCO_3_; 0.0147 g of CaCl_2_ 2H_2_O; 0.084 g of NaHCO_3_; 0.4036 g of NaCl, 0.00746 g of KCl in 50 mL mili-Q water, adjusting pH to 7.4 with 3N HCl). The analyses were performed within 24 h following blood collection. Nonetheless, some aliquots could not be analyzed due to conservation problems, but this did not unbalance sample sizes of CAR and diquat treatments (all χ^2^ tests: *P* > 0.10). Eighty microliters of KRL-diluted blood were incubated at 40 °C with 136 μL of a 150 mM solution of AAPH. The lysis of red blood cells was assessed with a microplate reader device (PowerWave XS2, Bio-Tek Instruments Inc., Winooski, VT), which measures the decrease in optical density at the wavelength of 540 nm every few minutes. Blood samples of a different bird species (zebra finch, *Taeniopygia guttata*) assessed twice were repeatable (*r* = 0.84, *P* < 0.001, *n* = 43). Units are reported as minutes.

### Plasma antioxidants

The total antioxidant status (TAS) of blood plasma was analyzed to estimate the availability of circulating hydrosoluble antioxidants. Since the idea that this measure assesses all the antioxidants is questionable, the term “total” was avoided, and hence, we will only use the generic “Plasma Antioxidants” (PLAOX). The procedure is based on Miller et al. (1993) modified by Cohen, Klasing & Ricklefs (2007) and Romero-Haro & Alonso-Alvarez (2014). Repeatability calculated on other samples of red-legged partridges assessed twice was high (*r* = 0.94, *P* < 0.001, *n* = 20; Galván & Alonso-Alvarez, 2009).

### Plasma biochemistry

Albumin, uric acid, triglycerides, LDL-cholesterol and total cholesterol levels in plasma were determined with commercial kits (Biosystems SA, Barcelona, Spain) with an automated spectrophotometer (A25-Autoanalyzer; Biosystems SA, Barcelona, Spain). The last three parameters are components of lipoproteins that act as carotenoid carriers in blood (McGraw & Parker, 2006). They were assessed to test for differences in lipid absorption due to direct diquat effects on the gut (see also Alonso-Alvarez & Galván, 2011), but the diquat factor or its interaction with the CAR factor did not provide any significant influence on their levels (all *P*-values > 0.16).

### Lipid peroxidation

The measurement of lipid peroxidation in plasma, liver and heart was carried out following the method described in Romero-Haro & Alonso-Alvarez (2014). Livers and hearts were previously diluted (1:10 w/v) and were homogenized with a stock buffer (phosphate buffer 0.01 M adjusted to pH 7.4 with HCl 37%). Aliquots of 50 μL of the samples (plasma, homogenized liver and heart samples, and standards) were then capped and vortexed for 5 s, and were analyzed as described in Romero-Haro & Alonso-Alvarez (2014). Zebra finch plasma samples assessed twice provided very high within-session (*r* = 0.97, *n* = 20, *P* < 0.001) and between-session (*r* = 0.98, *n* = 20, *P* < 0.001) repeatabilities (Romero-Haro & Alonso-Alvarez, 2014).

### Statistical analyses

All the analyses were performed using SAS v9.3 software (SAS Institute, Carry North Carolina, USA). The analyses are organized in two parts: (1) one testing the influence of carotenoid supplements only, and (2) the second analyzing the impact of the oxidative challenge (diquat exposure) and its interaction with carotenoid treatments.

The treatment effects on the number of birds producing eggs were calculated from contingency tables (*χ*^2^). These analyses were separately performed for each experimental period (carotenoid exposure only or diquat exposure) and sex. Sex was considered because some females escaped during the experiment and hence sample sizes differed between sexes (see above). The variability in the number of eggs per individual was tested using a GENMOD procedure in the SAS software, including the number of eggs as a multinomial variable with cumulative logit link.

To test the carotenoid treatment (CAR hereafter) effect on color and blood variables throughout the study (i.e., three different measures), repeated-measures mixed models (PROC MIXED in SAS; Littell et al., 2006) were used. In these models, the sampling event (TIME hereafter) was included as the repeated-measures factor, whereas the identity of the individual nested into cage identity was the subject term (REPEATED statement; Littell et al., 2006). CAR (four-level factor), TIME (three-level factor) and sex were always included in the models as fixed effects, testing their two- and three-way interactions. Since the aim was exclusively testing the CAR effect with the highest available statistical power, these repeated-measure models did not include data from those individuals exposed to diquat (day 82 only).

To analyze the effect of diquat, variability at the last sampling (day 82) was analyzed by generalized mixed models (PROC MIXED in SAS). Here, CAR and diquat treatments and sex were tested as fixed factors, testing their interactions. Color and blood levels at the precedent sampling event (day 48) were tested as covariates to correct for subtle differences between groups at the start of the diquat exposure (see section Variability after diquat exposure).

Other different covariates were added to the models. Thus, as previously mentioned, the redness (inverse of hue) of the eye ring and bill was controlled for total brightness. In the case of the leg, the redness of the red chip was tested. In all the repeated-measures mixed models testing the CAR effect, the influence of plasma vitamin (tocopherol and retinol) levels was tested by including them as covariates. In all the mixed models testing the diquat effect, plasma vitamin levels in the last sampling event, as well as vitamin levels in every internal tissue and ornaments, were also added. In models testing plasma MDA values, plasma triglyceride levels were added to control for potential influences of lipid variability in the blood (Romero-Haro & Alonso-Alvarez, 2014; Romero-Haro, Canelo & Alonso-Alvarez, 2015). In models testing PLAOX, uric acid, and albumin values were simultaneously tested to control for influences of recent food intake (Cohen, Klasing & Ricklefs, 2007). To control for subtle differences in reproductive investment, the number of eggs produced at the end of each sampling interval (“eggs”) was also tested as a covariate in repeated models (Table 2). In models testing final variability (Tables 3 and 4), the total number of eggs at the end of the study or the number of eggs during only the diquat experiment were tested as alternative covariates (in different models). The lag time (min) to start hemolysis and hematocrit were added as covariates in models testing resistance to hemolysis. Finally, the identity of the bird nested into the identity of the cage and the laboratory session were included as random factors (*P*-values ranging from < 0.001 to 0.476).

**Table 2:** Mixed models testing the interaction between carotenoid treatment and time. The reported tests are the best fitted models with an interaction at *P* < 0.10, or instead, when it is removed at higher *P*-values by following a backward-step wise procedure (see Methods).

Dependent variable	Terms in the model	Slope	SE	F	df	P
Eye rings redness	Carotenoid			10.22	3,167	<0.001
	Sex			3.97	1,167	0.048
	Time			5.43	2,237	0.005
	Carotenoid × time			2.65	6,237	0.017
	Eggs	−0.001	0.001	5.05	1,237	0.026
	Total brightness	−0.0001	0.0001	18.05	1,237	<0.001
	Plasma tocopherol	0.026	0.009	7.98	1,237	0.005
Bill redness	Carotenoid			6.58	3,168	<0.001
	Time			8.64	3,235	<0.001
	Carotenoid × time			1.96	6,235	0.072
	Eggs	−0.001	0.001	5.87	1,235	0.016
	Total brightness	−0.0002	0.00002	63.68	1,235	<0.001
	Plasma tocopherol	0.033	0.011	9.23	1,235	0.003
Legs redness	Carotenoid			6.04	3,167	<0.001
	Sex			17.6	1,167	<0.001
	Time			12.44	2,227	<0.001
	Sex × time			1.36	2,227	0.258
	Carotenoid × time			0.63	6,227	0.703
	Eggs	−0.025	0.022	1.28	1,227	0.259
	Red chip	1.251	0.274	20.85	1,227	<0.001
	Plasma tocopherol	1.129	0.467	5.84	1,227	0.017
	Plasma retinol	−1.854	1.147	2.61	1,227	0.108
Plasma lutein	Carotenoid			105.1	3,164	<0.001
	Sex			54.22	1,164	<0.001
	Time			69.61	2,237	<0.001
	Sex × carotenoid			5.22	3,164	0.002
	Carotenoid × time			36.81	6,237	<0.001
	Plasma tocopherol	0.456	0.026	313.86	1,237	<0.001
	Plasma retinol	0.168	0.065	6.74	1,237	0.01
	Eggs	−0.004	0.001	8.57	1,237	0.004
Plasma zeaxanthin	Carotenoid			309.6	3,164	<0.001
	Sex			47.35	1,164	<0.001
	Time			73.54	2,235	<0.001
	Carotenoid × time			95.93	6,235	<0.001
	Sex × carotenoid			3.68	3,164	0.013
	Sex × time			4.4	2,235	0.013
	Plasma tocopherol	0.379	0.026	216.34	1,235	<0.001
	Plasma retinol	0.188	0.064	8.72	1,235	0.004
	Eggs	−0.006	0.001	20.9	1,235	<0.001
Plasma tocopherol	Carotenoid			2.61	3,167	0.053
	Sex			2.56	1,167	0.112
	Time			117.68	2,236	<0.001
	Carotenoid × time			2.63	6,236	0.017
	Sex × time			4.89	2,236	0.008
	Plasma retinol	0.548	0.122	20.25	1,236	<0.001
	Eggs	−0.009	0.002	14.94	1,236	<0.001
Plasma retinol	Carotenoid			2.11	3,168	0.101
	Time			36.06	2,238	<0.001
	Carotenoid × time			1.01	6,238	0.421
	Plasma tocopherol	0.0852	0.019	20.8	1,238	<0.001
	Eggs	−0.002	0.001	5.14	1,238	0.024
UA & ALB-corrected PLAOX	Carotenoid			7.19	3,164	<0.001
	Time			0.38	2,187	0.686
	Carotenoid × time			2.57	6,187	0.021
	Uric acid	0.768	0.053	2.14	1,187	<0.001
	Albumin	−0.498	0.112	19.99	1,187	<0.001
	Plasma retinol	0.228	0.098	5.17	1,187	0.024
Plasma TRG-corrected MDA	Carotenoid			1.05	3,164	0.371
	Sex			0.29	1,164	0.593
	Time			14.43	2,228	<0.001
	Carotenoid × time			0.71	6,228	0.645
	Sex × carotenoid			1.8	3,164	0.149
	Sex × time			0.78	2,228	0.46
	Plasma tocopherol	−0.04	0.045	0.81	1,228	0.37
	Plasma retinol	−0.089	0.108	0.67	1,228	0.413
	Plasma triglycerides	0.298	0.031	90.13	1,228	<0.001
	Eggs	0.001	0.002	0.37	1,228	0.543
Resistance to oxidative stress in erythrocytes	Carotenoid			1.48	3,163	0.223
	Sex			1.3	1,163	0.256
	Time			1.7	2,203	0.185
	Carotenoid × time			0.69	6,203	0.657
	Sex × carotenoid			0.59	3,163	0.619
	Plasma retinol	−20.202	8.811	5.26	1,203	0.023
	Eggs	−0.339	0.18	3.55	1,203	0.061
	Lag time	−0.122	0.021	35.9	1,203	<0.001

**Note:**

ALB, albumin; MDA, malondyaldehydes; PLAOX, plasma antioxidants; TRG, tryglycerides; UA, uric acid.

**Table 3:** Mixed models testing how the exposure to oxidative stress (diquat) interacted with the dietary carotenoid treatment at the end of the experiment. The level of each dependent variable in the sampling event precedent to the diquat exposure is included as a covariate for color and blood variables. The models describe the backward step (using the *P* = 0.10 threshold) previous to remove the diquat × CAR interaction (i.e. when it was non-significant; Methods).

Dependent variable	Terms in the model	Slope	SE	F	df	P
Eye rings redness	Carotenoid			24.93	3,147	<0.001
	Diquat			0.03	1,146	0.859
	Sex			0.8	1,146	0.373
	Carotenoid × diquat			1.38	3,146	0.252
	Sex × diquat			3.21	1,146	0.075
	Total brightness	−0.00004	0.00002	6.25	1,147	0.014
	Eye ring redness in day 48	0.291	0.064	20.95	1,147	<0.001
	Liver vitamin A	0.0004	0.0003	1.33	1,147	0.251
	Eye ring tocopherol	0.025	0.012	4.43	1,146	0.037
	Eggs during diquat experiment	−0.002	0.0008	4.44	1,146	0.037
Bill redness	Carotenoid			17.19	383.2	<0.001
	Diquat			4.23	176.6	0.043
	Sex			1.46	175.9	0.231
	Carotenoid × diquat			1.03	374.3	0.382
	Sex × carotenoid			1.34	369.7	0.269
	Total brightness	−0.0001	0.00003	23.14	1,143	<0.001
	Bill redness in day 48	0.151	0.056	7.4	1,134	0.007
	Bill tocopherol	0.048	0.014	11.7	1,118	<0.001
	Liver vitamin A	0.001	0.0004	5.72	1,138	0.018
Leg redness	Carotenoid			10.55	3,136	<0.001
	Diquat			0.23	1,137	0.631
	Sex			3.98	1,138	0.048
	Carotenoid × diquat			0.67	3,137	0.575
	Sex × diquat			0.33	1,137	0.567
	Sex × carotenoid			1.69	3,137	0.173
	Red chip	1.018	0.221	21.28	128.8	<0.001
	Leg redness in day 48	0.594	0.065	82.46	1,137	<0.001
	Leg tocopherol	2.752	0.605	20.73	1,137	<0.001
	Liver tocopherol	−1.469	0.544	7.28	1,138	0.008
	Liver vitamin A	−0.024	0.015	2.73	1,135	0.101
	Total number of eggs	−0.032	0.014	5.1	1,137	0.026
Total astaxanthin in the eye rings	Carotenoid			51.64	3,146	<0.001
	Diquat			1.47	1,151	0.227
	Carotenoid × diquat			3.21	3,147	0.025
	Sex			17.4	1,148	<0.001
	Sex × carotenoid			3.21	3,146	0.025
	Tocopherol in the eye rings	0.674	0.06	125.22	1,149	<0.001
	Total number of eggs	−0.004	0.002	6.54	1,145	0.012
Total papilioerythrinone in the eye rings	Carotenoid			19.9	388.3	<0.001
	Diquat			0.55	180.1	0.46
	Sex			13.83	179.6	<0.001
	Carotenoid × diquat			0.33	377.4	0.804
	Sex × diquat			1.74	181.8	0.19
	Sex × carotenoid			3.71	379.5	0.015
	Fat retinol	0.032	0.031	1.04	1,136	0.309
	Plasma tocopherol	0.3	0.182	2.71	1,140	0.102
	Tocopherol in the eye rings	0.85	0.149	32.42	1,140	<0.001
	Total number of eggs	−0.007	0.004	3.03	183.8	0.086
Tocopherol in the eye rings	Carotenoid			3.64	371.7	0.017
	Diquat			1.22	175.7	0.272
	Carotenoid × diquat			2.63	373.5	0.056
	Total number of eggs	−0.006	0.002	8.96	171.1	0.004
Total astaxanthin in the bill	Carotenoid			141.3	3,151	<0.001
	Sex			5.43	1,155	0.021
	Diquat			4.68	1,157	0.032
	Carrotenoid × diquat			2.67	3,155	0.049
	Plasma tocopherol	1.176	0.061	371.2	1,158	<0.001
	Total number of eggs	−0.007	0.002	17.86	1,151	<0.001
Total papilioerythrinone in the bill	Carotenoid			134.5	364.8	<0.001
	Diquat			0.08	168.8	0.774
	Sex			2.66	175.8	0.107
	Carotenoid × diquat			1.76	366	0.163
	Tocopherol in the bill	1.536	0.133	133.35	1,134	<0.001
	Plasma retinol	−9.373	4.689	4	1,140	0.048
	Total number of eggs	−0.009	0.003	9.17	173.4	0.003
Tocopherol in the bill	Carotenoid			2.94	3,158	0.035
	Diquat			3.91	1,162	0.05
	Carotenoid × diquat			3.09	3,160	0.029
	Sex			5.6	1,161	0.019
	Total number of eggs	−0.007	0.002	9.66	1,159	0.002
Total astaxanthin in the legs	Carotenoid			7.36	392.1	<0.001
	Diquat			0.13	178.8	0.7168
	Sex			2.98	186.9	0.088
	Carotenoid × diquat			0.07	376	0.974
	Sex × diquat			0.14	176.6	0.712
	Sex × carotenoid			0.56	374.3	0.645
	Plasma tocopherol	0.146	0.113	1.66	1,144	0.199
	Liver vitamin A	0.004	0.002	3.32	1,133	0.071
	Fat retinol	−0.032	0.018	3.19	1,130	0.077
	Tocopherol in the legs	0.526	0.097	29.25	1,141	<0.001
	Total number of eggs	0.003	0.002	1.37	187.5	0.244
Total papilioerythrinone in the legs	Carotenoid			4.17	392.9	0.008
	Diquat			0.61	184.9	0.436
	Sex			6.93	191.3	0.01
	Carotenoid × diquat			0.17	382.1	0.919
	Sex × diquat			0.24	181.1	0.627
	Sex × carotenoid			0.16	378.8	0.919
	Tocopherol in the legs	0.867	0.147	34.64	1,143	<0.001
	Liver vitamin A	−0.003	0.004	0.49	1,144	0.484
Tocopherol in the legs	Carotenoid			4.09	382.2	0.009
	Diquat			2.4	183.8	0.125
	Carotenoid × diquat			1.21	182.4	0.3126
	Sex			5.98	189.5	0.016
	Total number of eggs	−0.004	0.002	4.41	182.1	0.039
Plasma lutein	Carotenoid			151.01	3,149	<0.001
	Diquat			0.01	1,149	0.925
	Carotenoid × diquat			2.84	3,149	0.04
	Lutein at day 48	0.446	0.062	51.81	1,149	<0.001
	Plasma tocopherol	0.479	0.0326	215.52	1,149	<0.001
	Eggs during diquat experiment	−0.003	0.002	4.42	1,149	0.037
Plasma zeaxanthin	Carotenoid			321.37	3,146	<0.001
	Diquat			0.83	1,146	0.363
	Carotenoid × diquat			1.57	3,146	0.2
	Zeaxanthin at day 48	0.307	0.07	19.24	1,146	<0.001
	Plasma tocopherol	0.572	0.039	219.4	1,146	<0.001
	Fat tocopherol	−0.037	0.013	8.24	1,146	0.005
	Liver vitamin A	0.002	0.001	4.79	1,146	0.03
	Eggs during diquat experiment	−0.004	0.002	4.05	1,146	0.046
Plasma tocopherol	Carotenoid			5.26	395.1	0.002
	Diquat			2.72	182.3	0.103
	Sex			0.14	186.2	0.71
	Carotenoid × diquat			0.7	379.3	0.552
	Sex × diquat			0.7	182.7	0.404
	Tocopherol at day 48	0.199	0.069	8.31	1,139	0.005
	Liver vitamin A	−0.004	0.002	3.99	1,134	0.048
	Fat retinol	0.03	0.016	3.34	1,138	0.07
	Plasma retinol	0.259	0.162	2.57	1,141	0.111
	Total number of eggs	−0.003	0.002	2.21	184.2	0.141
Plasma retinol	Carotenoid			1.36	380.5	0.262
	Diquat			1.54	180.3	0.218
	Sex			0.51	177.9	0.476
	Carotenoid × diquat			0.51	377.7	0.678
	Sex × diquat			0.31	178.7	0.579
	Sex × carotenoid			0.64	379.5	0.589
	Retinol at day 48	33.86	4.27	62.89	1,134	<0.001
	Plasma tocopherol	5.209	1.95	7.13	1,140	0.009
	Eggs during diquat experiment	−0.258	0.095	7.34	173.6	0.008
Liver lutein	Carotenoid			128.63	3,157	<0.001
	Diquat			0.06	1,157	0.811
	Carotenoid × diquat			1.49	3,157	0.22
	Plasma tocopherol	0.075	0.036	4.45	1,157	0.037
	Liver tocopherol	0.263	0.028	85.5	1,157	<0.001
Liver zeaxanthin	Carotenoid			315.42	3,151	<0.001
	Diquat			0	1,154	0.971
	Carotenoid × diquat			3.06	3,151	0.03
	Plasma tocopherol	0.1	0.046	4.69	1,151	0.031
	Liver tocopherol	0.341	0.04	74.08	140.3	<0.001
	Plasma retinol	0.003	0.001	3.6	1,153	0.06
Liver tocopherol	Carotenoid			12.77	3,161	<0.001
	Diquat			6.47	1,161	0.012
	Carotenoid × diquat			2.76	3,161	0.044
Liver vitamin A	Carotenoid			57.35	3,152	<0.001
	Diquat			0.04	1,154	0.834
	Sex			22.3	1,154	<0.001
	Carotenoid × diquat			0.47	3,152	0.707
	Sex × diquat			0.71	1,152	0.399
	Plasma tocopherol	−11.032	4.001	7.6	1,153	0.007
	Liver tocopherol	10.309	3.538	8.49	155.8	0.005
	Total number of eggs	−0.394	0.07	31.26	1,152	<0.001
Fat lutein	Carotenoid			12.87	3,147	<0.001
	Diquat			0.06	1,148	0.808
	Sex			0.13	1,147	0.716
	Carotenoid × diquat			0.21	3,148	0.89
	Sex × diquat			0.19	1,147	0.662
	Sex × carotenoid			1.26	3,147	0.292
	Fat tocopherol	1.288	0.181	50.69	1,142	<0.001
	Liver vitamin A	0.022	0.01	4.62	1,147	0.033
	Plasma retinol	−0.039	0.016	5.77	1,147	0.018
	Fat retinol	0.241	0.087	7.72	1,148	0.006
Fat zeaxanthin	Carotenoid			46.74	3,148	<0.001
	Diquat			0.16	1,148	0.687
	Sex			0.28	1,147	0.598
	Carotenoid × diquat			0.12	3,148	0.948
	Sex × diquat			0.88	1,148	0.349
	Sex × carotenoid			1.23	3,148	0.302
	Fat tocopherol	0.911	0.15	37.07	1,129	<0.001
	Liver vitamin A	0.019	0.01	4.55	1,148	0.035
	Plasma retinol	−0.023	0.014	3.01	1,148	0.085
	Fat retinol	0.203	0.073	7.84	1,148	0.006
Fat tocopherol	Carotenoid			0.57	395.7	0.638
	Diquat			1.3	182.8	0.257
	Sex			0.04	181.4	0.849
	Carotenoid × diquat			0.15	382	0.931
	Sex × diquat			0.31	181	0.578
	Sex × carotenoid			1.71	379.4	0.171
	Liver vitamin A	0.005	0.004	1.11	1,130	0.295
	Plasma retinol	−0.012	0.007	3.05	1,138	0.083
	Total number of eggs	−0.005	0.005	0.99	191.4	0.323
Fat retinol	Carotenoid			29.11	3,149	<0.001
	Diquat			0.18	1,148	0.671
	Sex			0.14	1,147	0.706
	Carotenoid × diquat			0.24	3,149	0.867
	Sex × diquat			0.05	1,149	0.816
	Sex × carotenoid			1.06	3,148	0.368
	Plasma tocopherol	0.64	0.535	1.43	1,149	0.234
	Liver tocopherol	0.136	0.428	0.1	1,149	0.752
	Fat tocopherol	−0.103	0.165	0.39	164.6	0.537
	Total number of eggs	−0.025	0.01	6.77	1,148	0.01
UA & ALB-corrected PLAOX	Carotenoid			3.4	379.9	0.022
	Diquat			0.37	171.4	0.543
	Sex			1.61	369.7	0.209
	Carotenoid × diquat			1.34	169.6	0.269
	Carotenoid × sex			0.33	362.6	0.805
	Diquat × sex			0.03	175.9	0.855
	Carotenoid × diquat × sex			2.85	361.3	0.045
	AOX at day 48	0.331	0.11	9.07	173.1	0.004
	Fat tocopherol	0.054	0.03	3.21	1,103	0.076
	Uric acid	0.056	0.005	119.03	1,101	<0.001
	Albumin	−0.009	0.005	3.73	1,104	0.056
	Liver vitamin A	−0.004	0.002	3.69	187.2	0.058
	Eggs during diquat experiment	−0.011	0.005	4.66	183.9	0.034
Plasma TRG-corrected MDA	Carotenoid			0.29	3,139	0.836
	Diquat			6.84	1,139	0.009
	Sex			4.8	1,140	0.03
	Carotenoid × diquat			0.86	3,139	0.466
	Diquat × sex			4.45	1,140	0.037
	TRG-corrected MDA at day 48	2.419	0.745	10.48	1,139	0.002
	Triglycerides	0.004	0.001	36.4	1,140	<0.001
	Eggs during diquat experiment	0.126	0.044	8.28	1,140	0.005
Liver MDA	Carotenoid			1.27	388	0.289
	Diquat			0.2	177.4	0.659
	Sex			22.76	180.4	<0.001
	Carotenoid × diquat			1.96	376.7	0.127
	Carotenoid × sex			1.21	375	0.311
	Diquat × sex			0.15	175.4	0.699
	Carotenoid × diquat × sex			4.65	376	0.005
	Liver vitamin A	0.0002	0.0001	7.07	1,138	0.009
	Plasma tocopherol	0.0003	0.0001	3.73	1,139	0.055
Heart MDA	Carotenoid			0.09	3,157	0.963
	Diquat			0.95	1,157	0.331
	Sex			2.75	1,157	0.099
	Carotenoid × diquat			1.79	3,157	0.151
Erythrocyte resistance to oxidative stress	Carotenoid			0.35	361.1	0.793
	Diquat			5.66	161.1	0.021
	Carotenoid × diquat			2.27	361.4	0.09
	Lag time	−0.229	0.035	44.06	1,121	<0.001

**Note:**

ALB, albumin; MDA, malondyaldehydes; PLAOX, plasma antioxidants; TRG, tryglycerides; UA, uric acid.

**Table 4:** Best fitted models obtained when the diquat × CAR interaction is removed at *P* > 0.10 after a backward stepwise procedure (see Methods). Heart MDA and fat tocopherol did not maintain any term (all *P* > 0.10).

Dependent variable	Terms in the model	Slope	SE	F	df	P
Eye rings redness	Carotenoid			25.55	3,154	<0.001
	Total brightness	−0.00004	0.00002	7.71	1,155	0.006
	Eye ring redness in day 48	0.286	0.064	19.99	1,155	<0.001
	Eye ring tocopherol	0.023	0.012	6.72	1,154	0.011
	Eggs during diquat experiment	−0.002	0.001	6.21	1,155	0.014
Bill redness	Carotenoid			16.22	385.7	<0.001
	Diquat			4.46	177.9	0.038
	Total brightness	−0.0001	0.00003	22.32	1,150	<0.001
	Bill redness in day 48	0.163	0.054	9.1	1,141	0.003
	Bill tocopherol	0.045	0.013	11.6	1,132	<0.001
	Liver vitamin A	0.0008	0.0004	4.26	1,141	0.041
Leg redness	Carotenoid			10.86	377.2	<0.001
	Sex			2.86	180.3	0.095
	Red chip	1.035	0.217	22.83	127.4	<0.001
	Leg redness in day 48	0.58	0.064	81.19	1,147	<0.001
	Leg tocopherol	3.022	0.589	26.36	1,146	<0.001
	Liver tocopherol	−1.78	0.523	11.59	1,147	<0.001
Total papilioerythrinone in the eye rings	Carotenoid			25.34	3,153	<0.001
	Sex			15.53	1,156	<0.001
	Tocopherol in the eye ring	0.953	0.14	46.43	1,158	<0.001
	Eggs (total)	−0.009	0.004	5.43	1,153	0.021
Total papilioerythrinone in the bill	Carotenoid			131.19	368.3	<0.001
	Sex			2.9	176.3	0.092
	Tocopherol in the bill	1.564	0.129	147.34	1,142	<0.001
	Plasma retinol	−9.038	4.666	3.75	1,145	0.055
	Eggs (total)	−0.009	0.003	8.37	177.9	0.005
Total astaxanthin in the legs	Carotenoid			9.4	377.9	<0.001
	Sex			10.56	187	0.002
	Tocopherol in the legs	0.564	0.081	48.28	1,159	<0.001
Total papilioerythrinone in the legs	Carotenoid			5.24	384.2	0.002
	Sex			7.39	186.3	0.008
	Tocopherol in the legs	0.866	0.139	38.79	1,152	<0.001
Tocopherol in the legs	Carotenoid			3.97	385.7	0.011
	Sex			5.74	189.2	0.019
	Eggs (total)	−0.004	0.002	4.93	185.4	0.029
Plasma zeaxanthin	Carotenoid			322.78	3,150	<0.001
	Zeaxanthin in day 48	0.31	0.07	19.6	3,150	<0.001
	Plasma tocopherol	0.561	0.038	217.91	3,150	<0.001
	Fat tocopherol	−0.038	0.013	8.72	3,150	0.004
	Liver vitamin A	0.002	0.001	5.35	3,150	0.022
	Eggs during diquat experiment	−0.004	0.002	4.06	3,150	0.046
Plasma tocopherol	Carotenoid			5.78	398.2	0.001
	Diquat			4.26	186.9	0.042
	Tocopherol in day 48	0.19	0.066	8.32	1,144	0.005
	Liver vitamin A	−0.003	0.001	2.89	1,147	0.091
	Fat retinol	0.034	0.016	4.81	1,147	0.029
Plasma retinol	Plasma tocopherol	5.608	2.058	7.43	1,153	0.007
	Eggs during diquat experiment	−0.278	0.112	6.18	178	0.015
Liver lutein	Carotenoid			130.26	3,161	<0.001
	Plasma tocopherol	0.071	0.035	3.98	1,161	0.048
	Liver tocopherol	0.263	0.028	87	1,161	<0.001
Liver vitamin A	Carotenoid			59.87	3,157	<0.001
	Sex			23.03	1,159	<0.001
	Plasma tocopherol	−11.396	3.947	8.34	1,157	0.004
	Liver tocopherol	9.738	3.418	8.12	158	0.006
	Total number of eggs	−0.396	0.069	32.78	1,157	<0.001
Fat lutein	Carotenoid			13.3	3,156	<0.001
	Fat tocopherol	1.29	0.175	54.41	1,153	<0.001
	Liver vitamin A	0.02	0.01	4.42	1,157	0.037
	Plasma retinol	−0.036	0.016	5.34	1,156	0.022
	Fat retinol	0.271	0.084	10.44	1,157	0.002
Fat zeaxanthin	Carotenoid			46.81	3,163	<0.001
	Fat tocopherol	0.952	0.143	44.45	1,146	<0.001
	Liver vitamin A	0.018	0.008	5.32	1,162	0.022
	Fat retinol	0.208	0.069	9.03	1,163	0.003
Fat retinol	Carotenoid			35.37	3,167	<0.001
	Total number of eggs	−0.026	0.01	8.62	1,165	0.004
Plasma TRG-corrected MDA	Diquat			7.04	1,145	0.009
	sex			5.13	1,147	0.025
	Diquat × sex			4.66	1,145	0.033
	TRG-corrected MDA in day 48	2.366	0.739	10.26	1,144	0.002
	Triglycerides	0.004	0.001	38.25	1,146	<0.001
	Eggs during diquat experiment	0.136	0.043	10.17	1,146	0.002
Resistance to oxidative stress in erythrocytes	Diquat			5.64	167.4	0.02
	Lag time	−0.242	0.034	49.91	1,128	<0.001

All the mixed models were explored from the saturated models. They firstly included all the covariates (although see alternative options above), fixed factors, and factor interactions. Alternative models were then tested by removing terms at *P* > 0.10 by following a backward-stepwise procedure. The last best-fitted model was also compared to alternatives using the Akaike Information Criteria (AIC), providing similar conclusions. When tested as dependent variables, carotenoids and vitamins were transformed with mathematical functions to attain a normal distribution. All carotenoids and tocopherol levels were log-transformed, whereas vitamin A levels in the liver were transformed by a square root. In subcutaneous fat, carotenoid and retinol levels were standardized into two blocks because some sample sessions gave particularly low values. Differences are always provided as least square means ± SE from models; that is, considering random factors and any term in the final model. Pair-wise comparisons were done by means of LSD post hocs. The description of interactions and their figures in the main text are restricted to tests reporting *P* < 0.10. Other models, figures and tables containing means and SD from raw data are described in Supplemental Information.

## Results

### Egg laying

The treatments did not affect the number of individuals producing eggs during the first (carotenoid supply only; all χ^2^ tests: *P* > 0.34) or second (diquat × carotenoid supply interaction; all χ^2^ tests: *P* > 0.86) part of the experiment. Similarly, the treatments did not influence the number of eggs produced during the first period (all χ^2^ tests: *P* > 0.65) or the total number of eggs laid during the whole study (all χ^2^ tests: *P* > 0.11). The addition of tocopherol or retinol values as covariates did not change any of these results. The tests on egg production reported similar results when including those males that were housed with new partners during the study (all tests: *P* > 0.10).

### Influence of carotenoid supplements throughout the three sampling events

**Figure 1:**
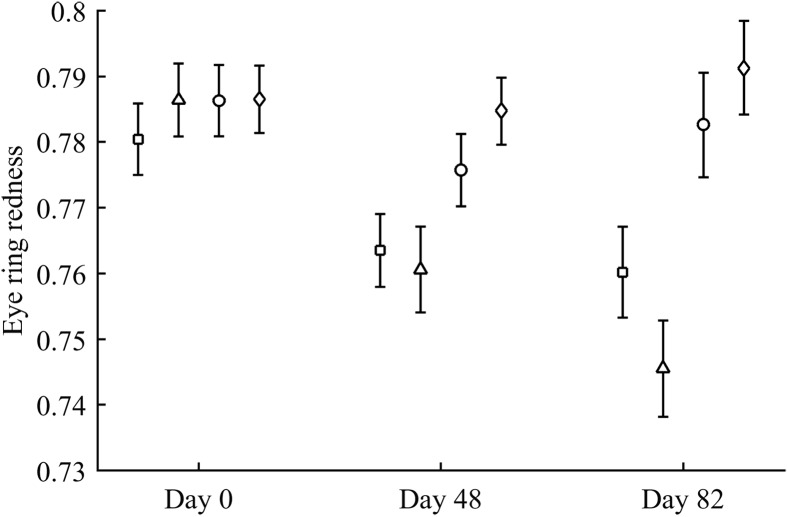
Changes in eye ring coloration during the experiment depending on the carotenoid treatment. Least square means ± SE were obtained from the models (see Methods). Squares: Control; Triangles: Astaxanthin; Circles: LutZea; Diamonds: ZeaLut.

Body mass was not affected by CAR treatments (time and sex interactions all *P* > 0.90). In contrast, integument coloration changed throughout the study according to carotenoid supplements. Redness decreased throughout reproduction, but the LutZea and ZeaLut groups counteracted this effect (CAR × time interaction) in the eye ring and bill, although the latter trait only showed a trend toward significance (Table 2; Fig. 1). In the eye ring, ZeaLut birds were redder than control and Ast partridges at the second sampling (both *P* < 0.05; Fig. 1). On the last day, LutZea and ZeaLut groups showed redder eye rings than the other treatments (*P* < 0.034), but did not differ between them (*P* = 0.411; Fig. 1). In the bill, differences arose at the last sampling, with LutZea, ZeaLut (both *P* < 0.001) and control (but *P* = 0.068) birds redder than Ast animals. ZeaLut and LutZea birds were also redder than controls, with the latter only a trend (*P* = 0.017 and 0.064, respectively; LutZea vs. ZeaLut: *P* = 0.673; Table 2; Fig. S1). The legs did not show a significant interaction (Table 2), although ZeaLut birds were redder than controls at the second and last samplings (both *P* < 0.013; Fig. S1).

**Figure 2:**
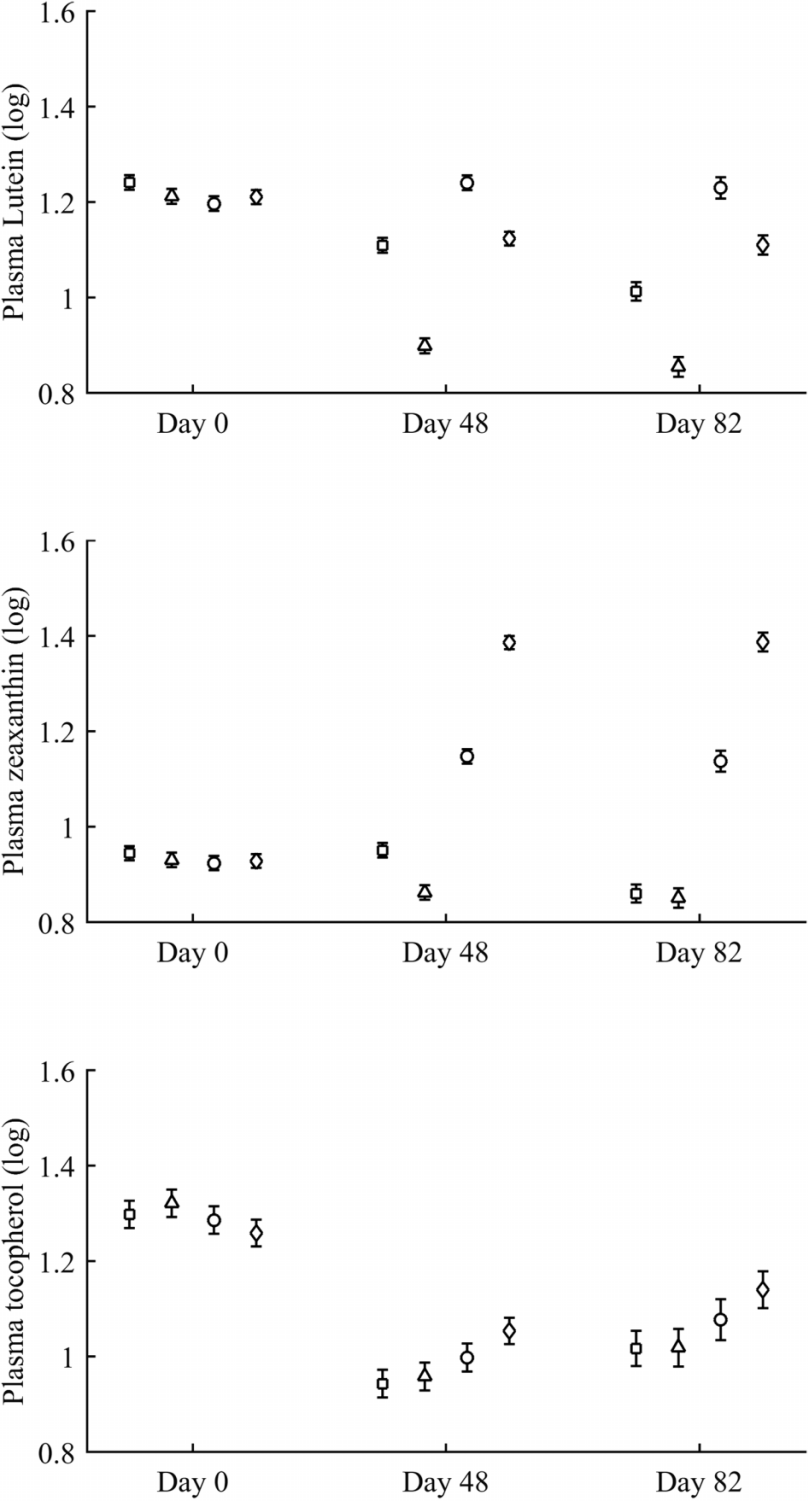
Changes in plasma carotenoid and tocopherol (log-transformed) levels during the experiment depending on the carotenoid treatment. Least square means � SE from the models (see Methods and Table 2). Squares: Control; Triangles: Astaxanthin; Circles: LutZea; Diamonds: ZeaLut.

In terms of plasma pigments, the carotenoid treatment interacted with time (Table 2; Fig. 2). Lutein levels did not differ between ZeaLut and control birds at day 48 (*P* = 0.48), but the other comparisons among groups on that day and at the last sampling were highly significant (all *P* < 0.001), with LutZea birds showing the highest values (Fig. 2). In the case of zeaxanthin, only Ast and control birds did not differ at the last sampling (*P* = 0.730), with the other groups differing clearly (all *P*-values < 0.001). Agreeing with predictions, ZeaLut partridges showed the highest zeaxanthin values (Fig. 2).

Plasma vitamins used as covariates in these models (Table 2) were also tested as dependent variables. The CAR × time interaction was not significant for retinol but was for tocopherol (Table 2; Fig. 2). ZeaLut birds showed higher tocopherol values than control and Ast individuals from 48 days to the end of the study (both *P* < 0.020; other comparisons: *P* > 0.13).

**Figure 3:**
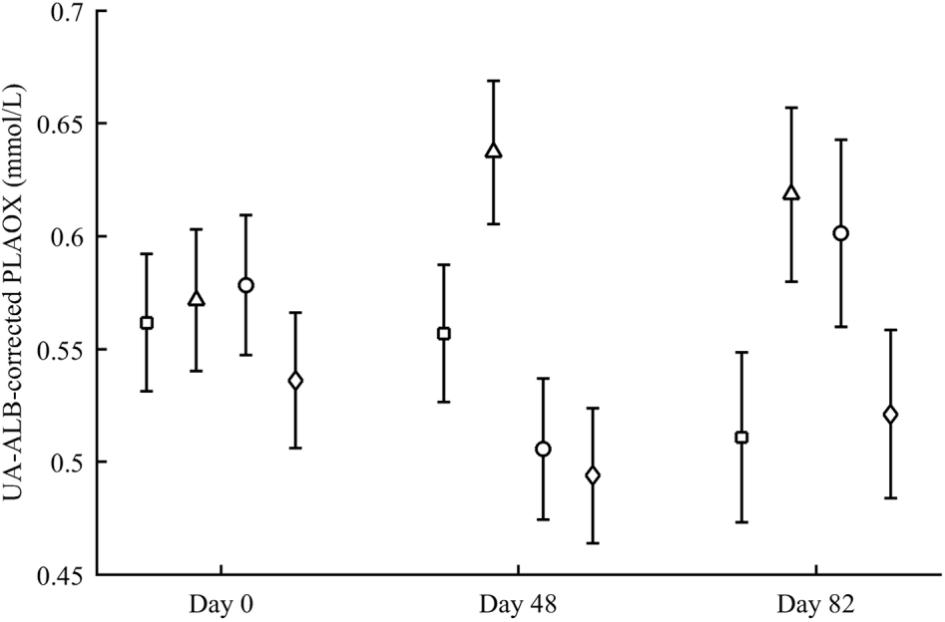
Changes in the levels (mmol/L) of plasma antioxidant status (controlled for albumin and uric acid levels) during the experiment depending on the carotenoid treatment. Least square means � SE from the models (see Methods and Table 2). Squares: Control; Triangles: Astaxanthin; Circles: LutZea; Diamonds: ZeaLut.

PLAOX changed according to the supplemented carotenoid (Table 2; Fig. 3). On day 48, Ast showed higher values than other groups (all *P* < 0.012), with controls reporting higher mean levels than ZeaLut (*P* = 0.034) and LutZea (but *P* = 0.098) birds. At the last sampling, LutZea birds increased their values approaching Ast individuals (*P* = 0.715). Ast birds again differed from the other two groups (both *P* < 0.023), with LutZea animals showing a trend toward higher values than control (*P* = 0.052) and ZeaLut (*P* = 0.080) birds. The interaction remained (*P* = 0.020) when removing albumin and uric acid covariates (Fig. 3).

Finally, plasma MDA (i.e., corrected or uncorrected for plasma lipid levels) and the resistance to oxidative stress in erythrocytes did not show significant differences with CAR during the study (all *P*-values > 0.64; Table 2).

### Variability after diquat exposure

When testing variables at the end of the study (i.e. after diquat exposure), body mass controlled for tarsus length variability was not influenced by CAR or diquat treatments or their interactions (all *P* > 0.10). The same was found for circulating LDL and total cholesterol levels (all *P* > 0.12).

#### Ornament color and pigments

**Figure 4:**
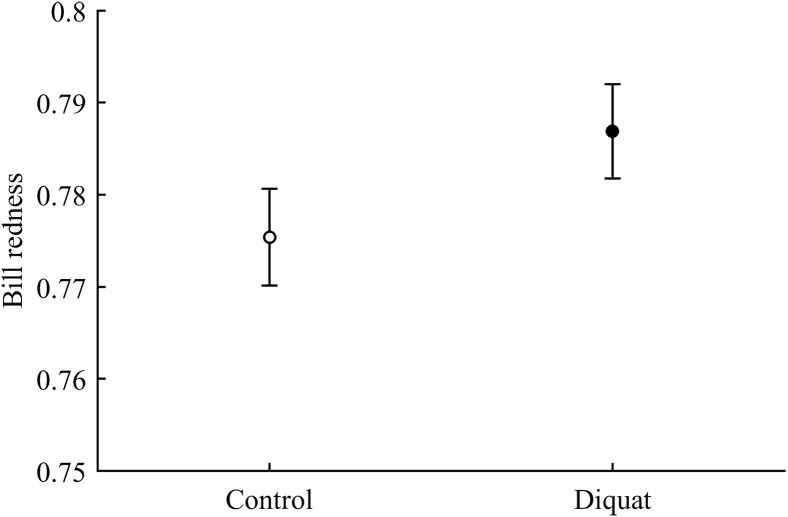
Effect of the diquat treatment on bill redness. Least square means ± SE from the models (see Methods and Table 4). Open circles: Control birds; Solid circles: Diquat-treated birds.

In terms of redness, CAR did not clearly interact with diquat in any trait (all *P* > 0.24; Table 3; Fig. S2). Nonetheless, diquat-exposed birds showed marginally significant redder bills among control and ZeaLut birds (*P* = 0.051 and 0.084, respectively; Fig. S2). Moreover, in the eye ring model, sex showed a trend toward a significant interaction with diquat (*P* = 0.069 in its last backward step). Males showed redder eye rings than females, but only among diquat-treated pairs (post hoc: *P* = 0.020; diquat male: 0.770 ± 0.006; diquat female: 0.757 ± 0.006; control male: 0.762 ± 0.006; control female: 0.764 ± 0.006; other pairwise comparisons: *P* > 0.18). In any event, in the best-fitted model excluding any interaction (i.e. Table 4), the diquat treatment alone reported a significant effect on bill redness, with diquat-treated birds showing redder bills (Fig. 4).

**Figure 5:**
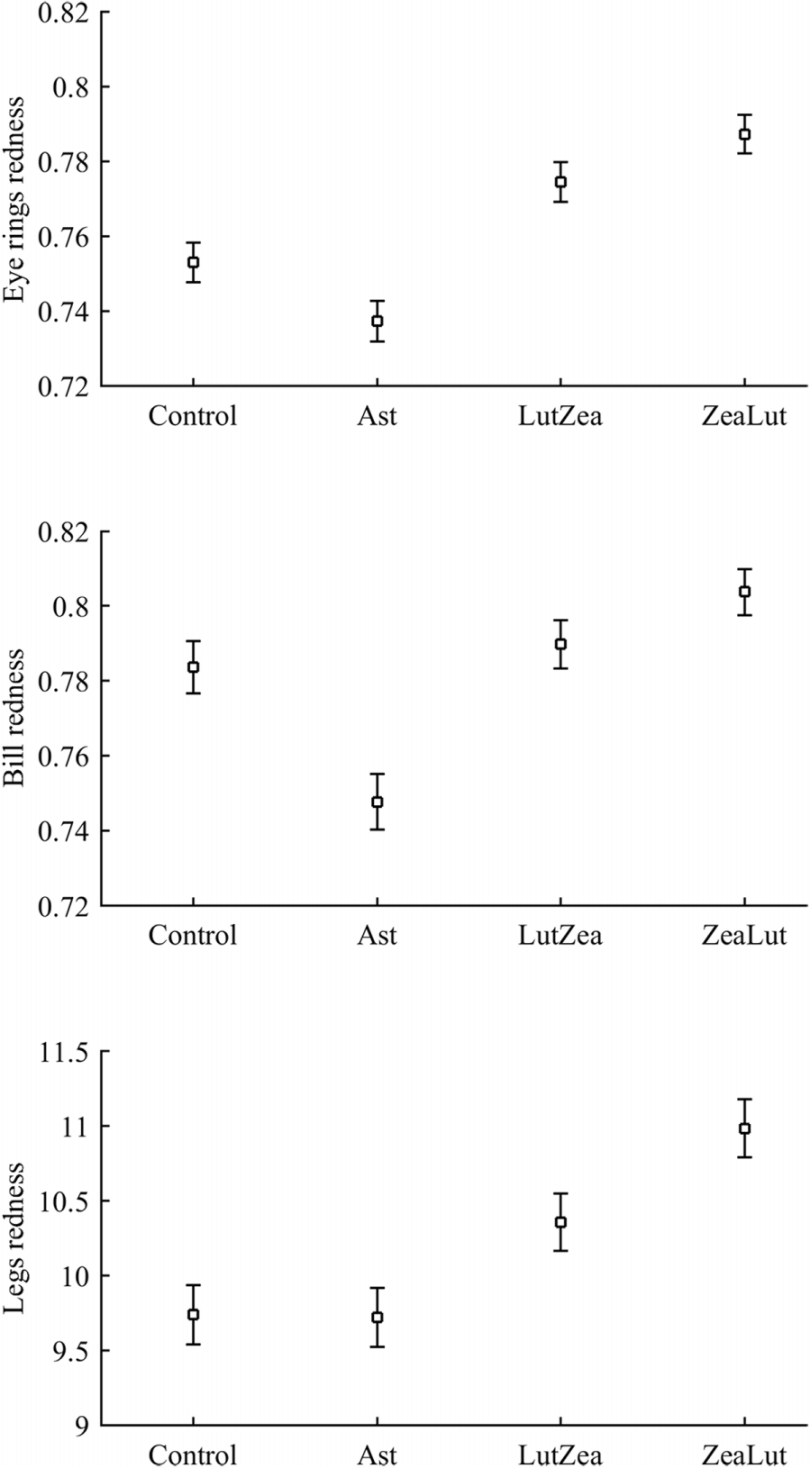
Final values of ornament coloration depending on carotenoid supplements exclusively. Least squared means � SE from the models controlling for the effect of the diquat treatment.

Best-fitted models for any ornament also showed a strong CAR effect (all *P*-values < 0.001; Table 4). Ast birds were always the palest individuals (all *P* < 0.001), whereas ZeaLut partridges were the reddest ones, followed by LutZea birds and controls (Fig. 5). Importantly, the difference in color between ZeaLut and LutZea animals was significant in eye rings and legs (both *P* < 0.044; in the bill: *P* = 0.065; Fig. 5).

**Figure 6:**
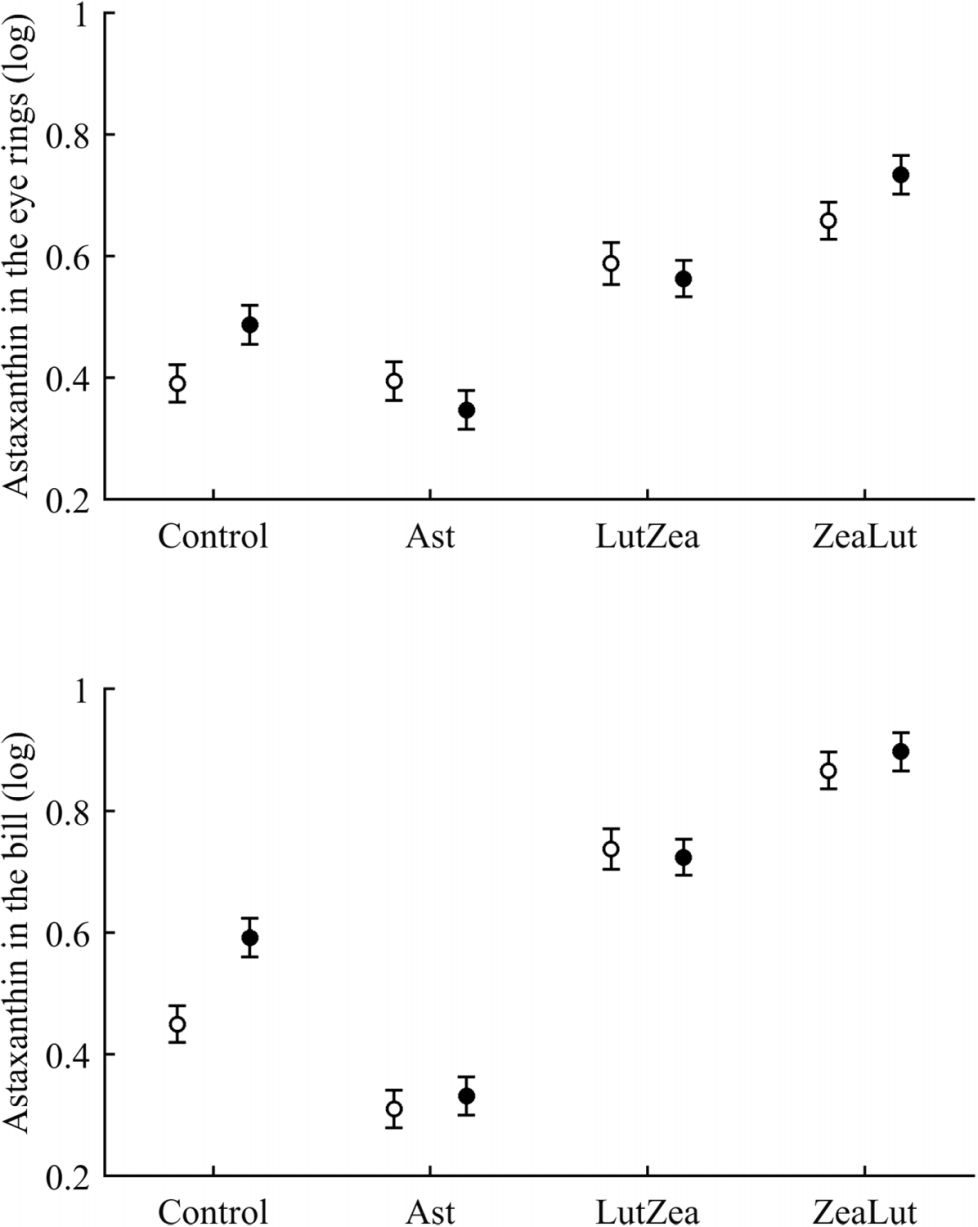
Levels of astaxanthin in the eye rings and bill after diquat exposure depending on the carotenoid treatment. Least square means ± SE from the models (see Methods and Table 3). Open circles: Control birds; Solid circles: Diquat-treated birds.

Concerning pigments, neither lutein nor zeaxanthin was detected. Diquat affected astaxanthin levels in the eye ring and bill but depending on CAR (Table 3; Fig. 6). The effect was partially due to differences in CAR-controls of both traits (both *P* < 0.020), with diquat-treated birds showing higher astaxanthin concentrations. Nonetheless, in the eye rings ZeaLut birds showed a marginally significant difference in the same direction (*P* = 0.057). In the same eye ring and bill models, all pair-wise comparisons between carotenoid groups (CAR factor: both *P* < 0.001) were significant (all *P* < 0.013), showing increasing astaxanthin values in the following order: Ast, control, LutZea and ZeaLut (Fig. 6). In legs, the diquat × CAR interaction did not affect astaxanthin (Table 3). Only CAR remained in the model (Table 4), with LutZea and ZeaLut birds showing higher astaxanthin levels (Fig. S3) than other groups (all *P* < 0.025), but not differing between them (*P* = 0.162; also Ast vs. control: *P* = 0.248).

In contrast to astaxanthin, papilioerythrinone was unaffected by diquat (any trait: *P* > 0.16; Table 3). The best-fitted model (Table 4) always reported a significant CAR influence (all traits: *P* < 0.010; Fig. S4). In the eye rings, LutZea and ZeaLut birds did not differ (*P* = 0.526), but other comparisons were significant (*P* < 0.012). In the bill, all CAR groups differed (*P* < 0.009), with LutZea showing higher levels than ZeaLut, and Ast showing the lowest values. In the legs, LutZea presented higher papilioerythrinone levels than other groups (all *P*’s < 0.017; differences among other groups *P* > 0.13; Fig. S4).

**Figure 7:**
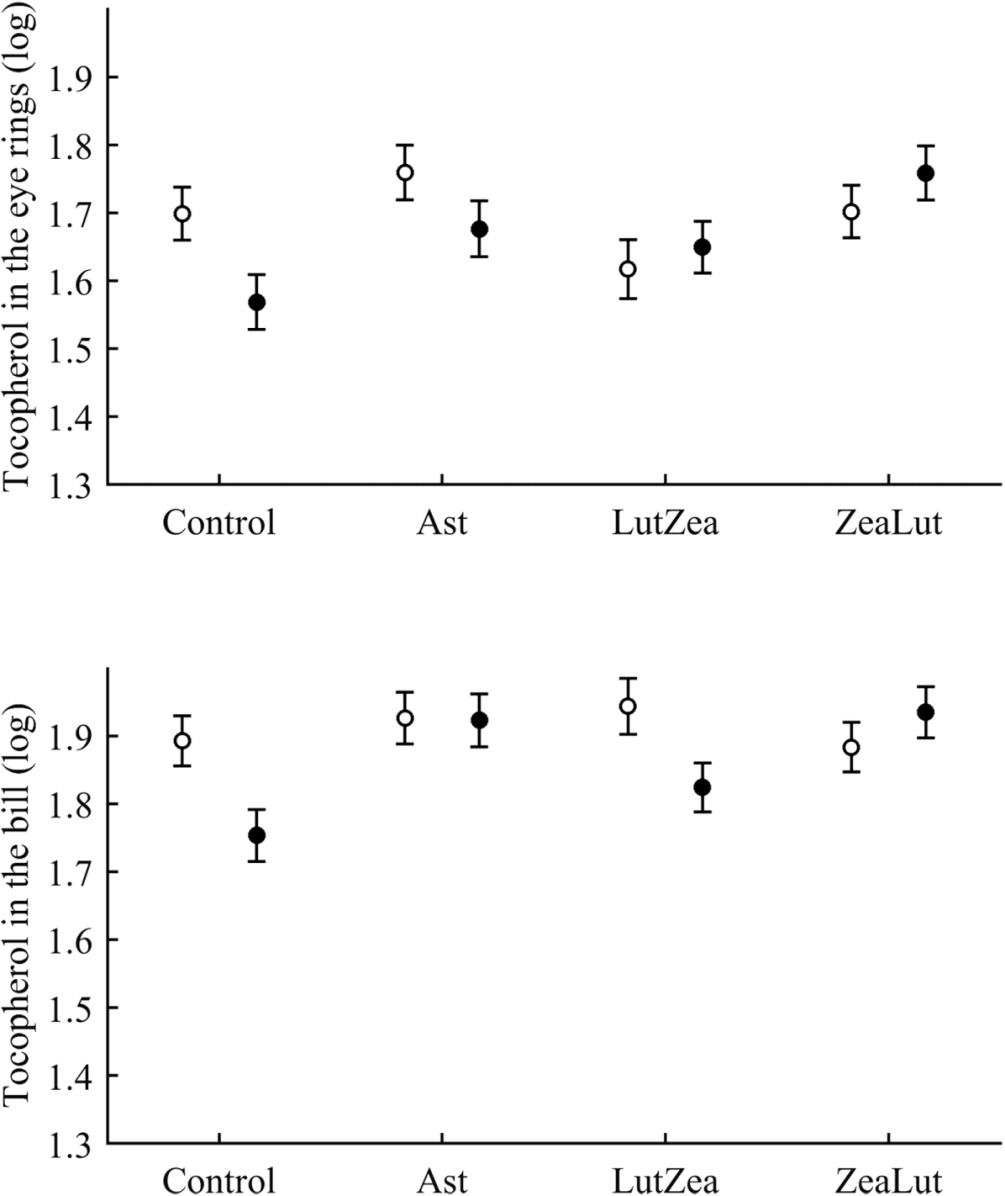
Levels of tocopherol in the eye rings and bill after diquat exposure depending on the carotenoid treatment. Least square means ± SE were obtained from the models (see Methods and Table 3). Open circles: Control birds; Solid circles: Diquat-treated birds.

Tocopherol and retinol are antioxidant vitamins whose variability could indirectly influence carotenoid values (although tested as covariates in the models; see Methods). Tocopherol, but not retinol, was detected in the ornaments. In the eye ring, the diquat × CAR interaction showed a trend toward significance (*P* = 0.056), with diquat decreasing tocopherol values in controls only (*P* = 0.021; Tables 3 and S2 for raw data; see also Fig. 7). In the same model, the CAR factor (*P* = 0.017) showed that ZeaLut partridges had higher tocopherol levels than LutZea and control birds (both *P* < 0.016), but Ast birds also showed higher vitamin levels than LutZea and control animals (both *P* < 0.039; other comparisons *P* > 0.75).

In the bill, tocopherol was also affected by diquat × CAR (Table 3; Fig. 7). Diquat decreased tocopherol values in control and LutZea individuals (both *P* < 0.05; Fig. 7). The CAR factor (*P* = 0.035) only indicated that controls had lower values than Ast and ZeaLut (both *P* < 0.020). Finally, only the CAR effect was significant in the legs (Tables 3 and 4). ZeaLut birds showed the highest tocopherol concentrations in the legs (both *P* < 0.005 when compared to LutZea and controls; *P* = 0.085 when compared to Ast).

#### Plasma and internal tissues

**Figure 8:**
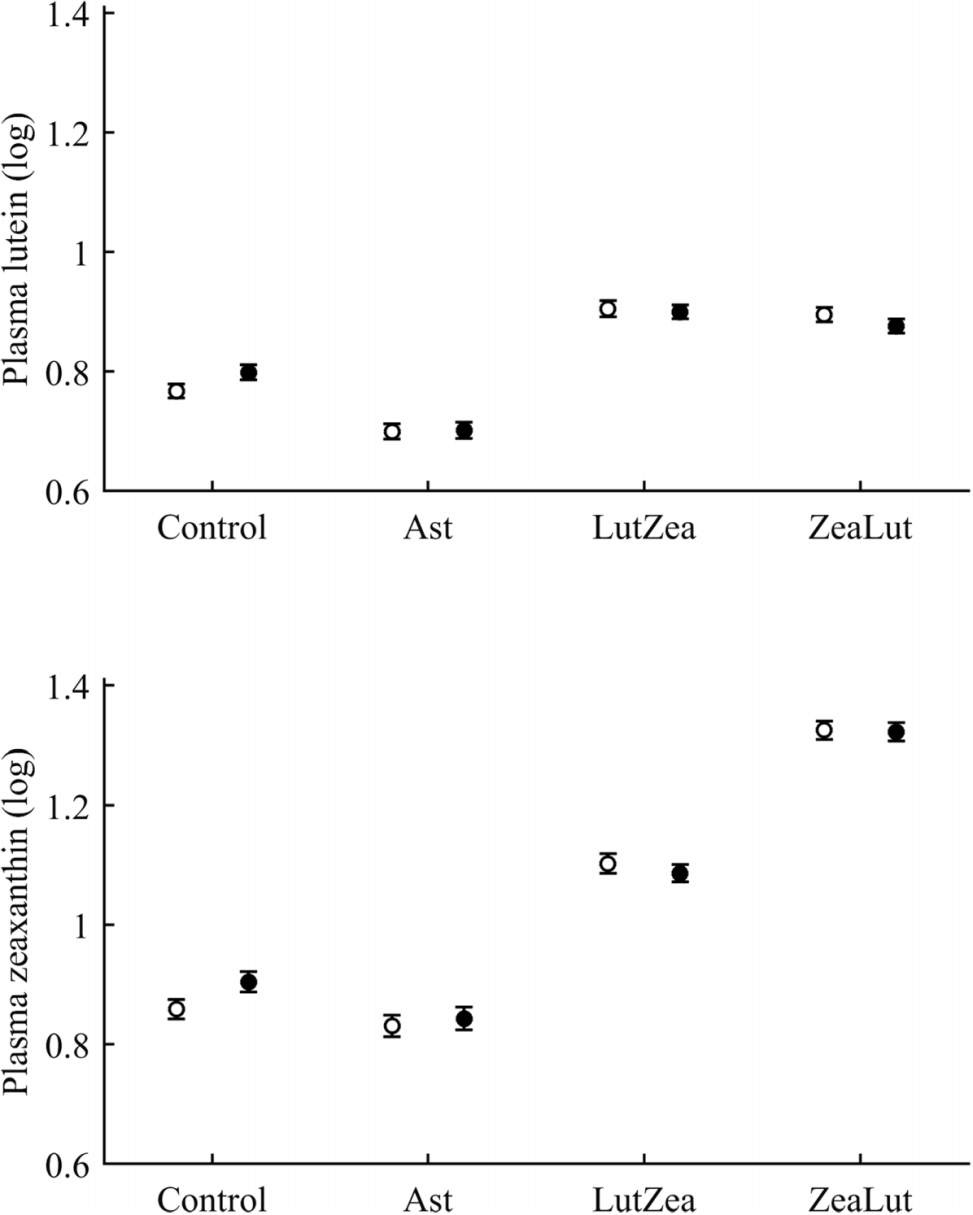
Levels of lutein and zeaxanthin in plasma after diquat exposure depending on the carotenoid treatment. Least square means ± SE were obtained from the models (see Methods and Table 3). Open circles: Control birds; Solid circles: Diquat-treated birds.

With regard to circulating carotenoids, lutein showed a significant diquat × CAR interaction (Table 3). Among CAR groups, only controls showed significantly higher lutein levels with diquat (*P* = 0.039; control: 0.98 ± 0.01; diquat: 1.02 ± 0.01, log-values; Fig. 8). In the case of zeaxanthin, although the CAR × diquat interaction was non-significant (*P* = 0.200; Table 3), the post hoc comparison within the control-CAR group showed a similar diquat effect (*P* = 0.033; control: 0.86 ± 0.02; diquat: 0.91 ± 0.02; Fig. 8). No other pigment showed detectable levels in plasma.

With regard to plasma vitamins, tocopherol was unaffected by the diquat × CAR interaction (Table 3). Nonetheless, diquat showed a significant effect (Table 4), with tocopherol values decreasing after the exposure (control: 1.08 ± 0.02; diquat: 1.03 ± 0.02). No factor or interaction was significant in the case of plasma retinol (all *P* > 0.10; Table 4).

**Figure 9:**
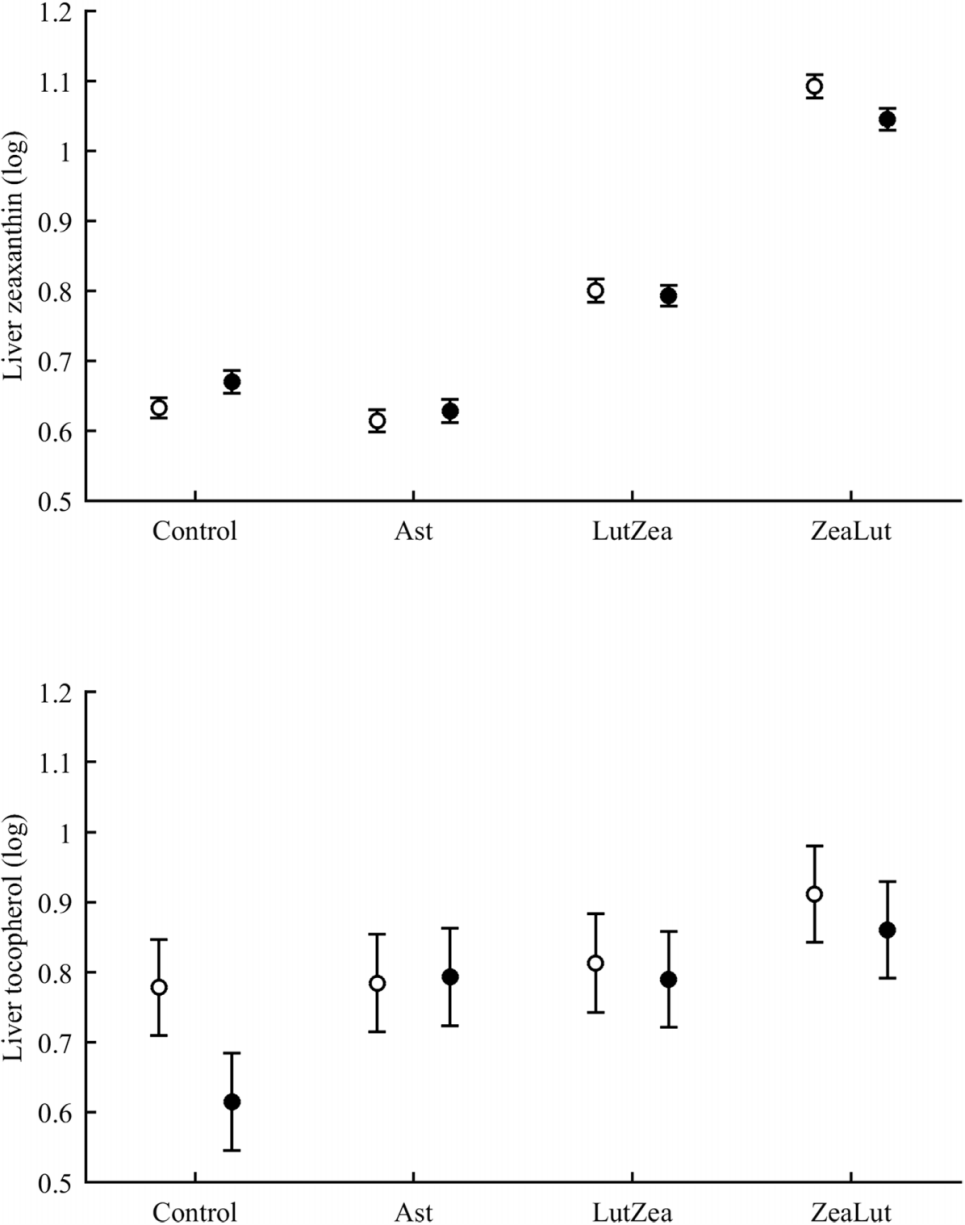
Levels of zeaxanthin and tocopherol in the liver after diquat exposure depending on the carotenoid treatment. Least square means ± SE were obtained from the models (see Methods and Table 3). Open circles: Control birds; Solid circles: Diquat-treated birds.

In the liver, the diquat × CAR interaction did not affect lutein levels (Table 3). The best-fitted model reported a strong significant CAR effect (Table 4). LutZea and ZeaLut birds did not differ (*P* = 0.103) and showed the highest lutein levels (Fig. S5). The other comparisons always reported *P* < 0.001, and the Ast group showed the lowest value (Table S3). In contrast, liver zeaxanthin showed a significant CAR × diquat interaction (Table 3). This effect was mostly due to diquat reducing zeaxanthin levels in ZeaLut birds (*P* = 0.028), and a trend in the opposite direction among controls (*P* = 0.064; Fig. 9). Importantly, such as in the case of astaxanthin in ornaments, the CAR factor (*P* < 0.001) reported increasing liver zeaxanthin values in the following order: Ast, control, LutZea and ZeaLut (all comparisons: *P* < 0.040).

With regard to liver vitamins, tocopherol was affected by CAR × diquat (Table 3). Among CAR groups, only control values showed a diquat effect on tocopherol, i.e. a decline (*P* = 0.001; Fig. 9). The CAR factor in the same model (*P* < 0.001) indicated significant differences following the order showed above for liver zeaxanthin (all *P* < 0.003), but here control and Ast birds did not differ (*P* = 0.699). In the case of liver retinol, both free and esterified retinol forms were detected, the two values being added for analyses (i.e. vitamin A). This variable was unaffected by CAR × diquat (Table 3) but showed a significant CAR effect (Table 4). LutZea and ZeaLut birds did not differ (*P* = 0.133), with Ast animals reporting the highest level, and control birds the lowest (other *P* < 0.001; Fig. S5).

In the subcutaneous fat, no carotenoid or vitamin was affected by CAR × diquat (all *P* values > 0.80; Table 3). The best-fitted models always reported a significant CAR effect (Table 4; Fig. S6; except for tocopherol). In the case of lutein, all groups differed from each other (all *P* < 0.001), except ZeaLut vs. control (*P* = 0.915). The LutZea group reported the highest lutein levels, and Ast birds the lowest. For zeaxanthin, LutZea birds tended to show higher values than controls (*P* = 0.062), with other groups significantly differing from each other (all *P* < 0.012). ZeaLut birds presented the highest zeaxanthin values, whereas Ast again showed the lowest. Tocopherol was not affected by any factor or interaction (all *P* > 0.10; Table 3; Fig. S7). With regard to retinol, all the groups differed from each other (CAR factor in Table 4), except ZeaLut and LutZea (*P* = 0.955). Ast and control birds showed the highest and lowest values, respectively (all *P* < 0.001; Fig. S6).

#### Oxidative stress biomarkers

**Figure 10:**
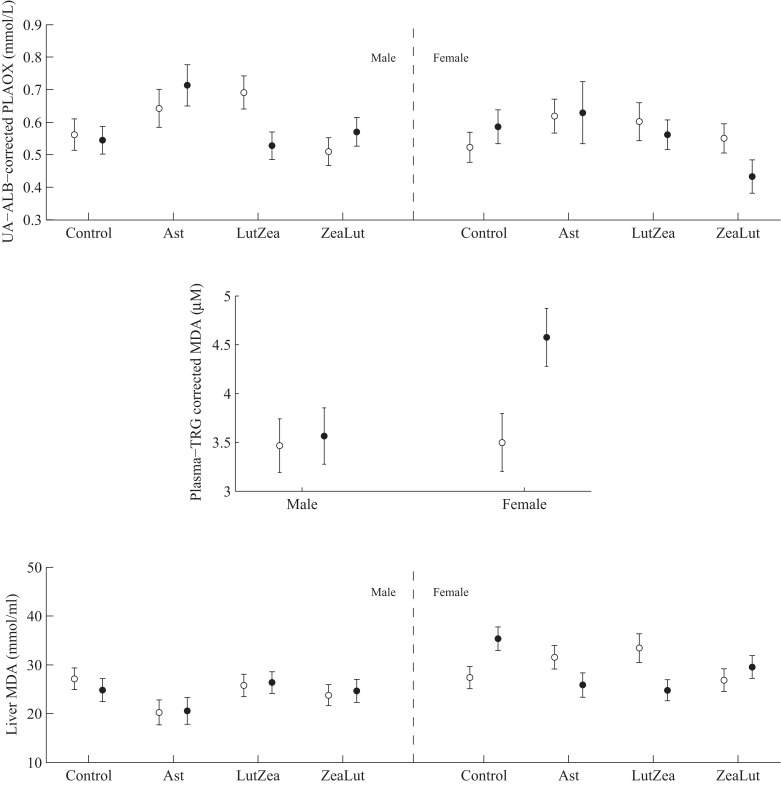
Levels of oxidative stress biomarkers after diquat exposure depending on the carotenoid treatment. Least square means ± SE from the models (see Methods and Table 3). Open circles: Control birds; Solid circles: Diquat-treated birds.

PLAOX showed a three-way CAR × diquat × sex interaction (Table 3; Fig. 10). Diquat decreased hydrosoluble antioxidant levels in LutZea males (*P* = 0.02), showing a trend in the same direction in females, but in the ZeaLut group (*P* = 0.06; Fig. 10). No factor or interaction remained (all *P* > 0.18) when removing uric acid and albumin covariates (though they showed *P* < 0.057).

In plasma MDA, CAR × diquat was non-significant (*P* = 0.466; Table 3), but diquat × sex interacted (Table 4; Fig. 10). Diquat-treated females showed higher lipid peroxidation than control females (*P* = 0.001; males did not differ: *P* = 0.752). The interaction did not change (*P* = 0.008) when removing the triglyceride covariate. The CAR group was never significant (*P* > 0.5). In liver MDA, the three-way interaction again arose (Table 3; Fig. 10). Diquat increased MDA values in control females (*P* = 0.009), but decreased MDA in LutZea (*P* = 0.014) and Ast (but at *P* = 0.079) females. Moreover, diquat control-CAR females also tended to endure higher liver MDA values than diquat ZeaLut females (*P* = 0.068). No difference was found in males (all *P* > 0.10). The CAR group in the model was not significant (*P* = 0.289). No factor or interaction reported significant terms in heart MDA (Table 4; all *P* > 0.12).

**Figure 11:**
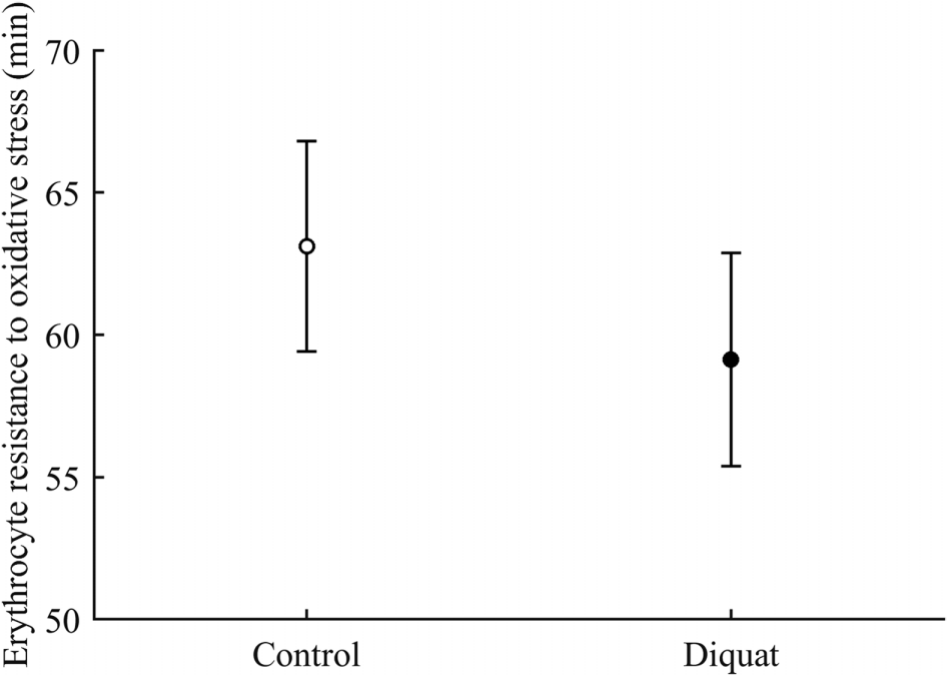
Effect of the diquat treatment on the erythrocyte resistance to oxidative stress. Least square means � SE from the models (see Methods and Table 4). Open circles: Control birds; solid circles: diquat birds.

Finally, in the case of erythrocyte resistance to oxidative stress, the CAR × diquat interaction only showed a weak trend toward significance (*P* = 0.090; Table 3), but the best-fitted model reported a significant diquat effect (Table 4; Fig. 11). The CAR factor was not significant (all *P* > 0.50).

## Discussion

Our results as a whole suggest that the availability of certain carotenoids in the diet and the level of oxidative stress can coexist and interact to produce pigmentation in avian ornaments. As predicted, a higher dietary content of lutein vs. zeaxanthin (LutZea) led to a higher papilioerythrinone accumulation in the red ornaments, whereas the opposite (ZeaLut) led to a higher astaxanthin deposition. Birds fed with higher zeaxanthin and lutein proportions showed the reddest ornaments, but the first (ZeaLut) showed the reddest traits (eye rings and legs) at the end of the study. Furthermore, the oxidative challenge produced redder bills and higher astaxanthin deposition in the bare parts of some birds, the latter depending on tocopherol levels in the same tissue.

### Covariation between vitamins and carotenoids

The carotenoid treatments affected tocopherol levels in tissues. However, this only partially agreed with diet composition, which showed the highest vitamin values in the ZeaLut and, particularly, the Ast groups (Table 1). In legs, plasma and liver, only ZeaLut birds showed higher tocopherol levels than other groups. The lack of high tocopherol values in Ast partridges in these tissues could be explained by astaxanthin interfering with vitamin absorption (e.g. Giraudeau et al., 2013; also below). Nonetheless, both Ast and ZeaLut groups showed the highest tocopherol levels in the other ornaments. In the case of retinoids, Ast birds also showed the highest value in liver and fat, but ZeaLut and LutZea groups did not differ.

Results also suggest that carotenoids protected vitamin E from oxidative stress. In the bill, eye rings, and liver, diquat decreased tocopherol levels, but only among birds that did not receive carotenoid supplements. This suggests that a higher carotenoid availability among CAR-treated birds buffered tocopherol consumption due to free radicals, which supports the idea of mutual recycling and protective roles between tocopherol and carotenoids (Mortensen, Skibsted & Truscott, 2001; Catoni, Peters & Schaefer, 2008; Surai, 2012). The only exception was the diquat-mediated reduction in tocopherol levels in the bill of LutZea birds. Regardless, we must consider that, among carotenoids, lutein (i.e. the most abundant carotenoid in LutZea birds) is the weakest antioxidant (Britton, 1995; Martínez et al., 2008; see also below).

To discriminate carotenoid effects from the influence of vitamin variability in the diet, all the statistical models were controlled for tocopherol and retinoid levels in the tissues. The problem of collateral variation of antioxidants in a supplemented diet has mostly been ignored in experiments aiming to strictly manipulate dietary carotenoid levels. For instance, Stirnemann et al. (2009), Toomey & McGraw (2011) and Toomey & McGraw (2012) have used the same beadlets including zeaxanthin and tocopherol, but vitamin levels were not considered in their analyses. The addition of other antioxidants as excipients in carotenoid supplements would protect carotenoids during storage. In the other direction, the addition of carotenoids to the pelleted food could also have protected antioxidant vitamins in the basal diet, thus disrupting the original covariation among the levels of different compounds (Table 2; Catoni, Peters & Schaefer, 2008). In any event, we must note that diquat effects within a particular carotenoid treatment were independent of vitamin variability in that group as both diquat and control birds should have received the same vitamin amounts. In summary, results must be carefully interpreted in the light of vitamin covariation.

### Metabolic pathway of dietary carotenoids

We predicted that birds supplemented with astaxanthin should produce the most pigmented ornaments as biotransformation is not required. Surprisingly, dietary astaxanthin was apparently not absorbed. It was not detected in blood and other internal tissues. Moreover, astaxanthin seems to have interfered with lutein, zeaxanthin and tocopherol acquisition, as the circulating levels of these molecules declined in Ast birds. Consequently, ketocarotenoid deposition in ornaments and trait redness were reduced. Carotenoid competition during intestinal absorption and/or incorporation into the chylomicrons (e.g. Tyssandier et al., 2002; Canene-Adams & Erdman, 2009) can be argued considering the literature on humans (reviewed in Furr & Clark (1997) and van den Berg (1999)). In birds, competitive interactions of beta-carotene vs. lutein or zeaxanthin during intestinal absorption have also been reported for poultry diets (Wang et al., 2010). Interestingly, in the opposite direction, flamingoes (*Phoenicopterus ruber*) fed with lutein or zeaxanthin were unable to absorb these two pigments, but were instead able to assimilate astaxanthin, which is used as a precursor for the main carotenoid in their feathers (i.e. canthaxanthin; Fox & McBeth, 1970; McGraw, 2006). Our partridges also differ from European storks (*Ciconia ciconia*) naturally feeding on crayfish (*Procambarus clarkii*) containing high astaxanthin concentrations because they showed redder skin and higher astaxanthin concentrations in blood than controls (Negro & Garrido-Fernández, 2000). Phylogenetic differences may explain this. Astaxanthin is common in waterbirds feeding on fishes and aquatic invertebrates (an important astaxanthin source), but not among other avian species (McGraw, 2006). The red-legged partridge is a terrestrial granivorous gallinacean, and thus, astaxanthin is probably infrequent in their natural diet. For this reason, the capacity for assimilating astaxanthin may not have evolved. Nonetheless, other granivorous (but passerine) birds are able to absorb canthaxanthin (McGraw & Hill, 2001; Hill, 2002), another carotenoid described in aquatic organisms (McGraw, 2006).

On the other hand, our manipulation mostly supports the biotransformation pathway proposed for red-legged partridge carotenoids (i.e. García-de Blas et al., 2014); that is, lutein acting as the main papilioerythrinone precursor, with zeaxanthin acting as the main astaxanthin substrate. Lutein and zeaxanthin levels rose in the blood, liver and fat according to their relative abundance in the diet. Similarly, papilioerythrinone and astaxanthin in ornaments increased in higher amounts in LutZea and ZeaLut groups, respectively. The results support previous correlative findings in the same species (García-de Blas, Mateo & Alonso-Alvarez, 2015) and demonstrate that the ketocarotenoids giving color to red-legged partridge ornaments are influenced by the availability of the most common hydroxycarotenoids in birds (McGraw, 2006). As previously mentioned, lutein and zeaxanthin are the most frequently described and abundant carotenoids in the food and blood of many bird species, as well as the most common substrates for red ketocarotenoids in ornaments, at least among non-aquatic species (Surai et al., 2001; McGraw, 2006). In passerines, lutein levels always prevail over zeaxanthin levels in both blood and diet, commonly at a 70:30 ratio (lutein:zeaxanthin) or higher (e.g. McGraw et al., 2004), which could also reflect the dietary content (McGraw, 2006). Our manipulation supports this for a gallinacean species. Moreover, McGraw et al. (2004) proposed that birds should prioritize zeaxanthin accumulation because this pigment would proportionally contribute more to coloring red ornaments compared to lutein. This has only been supported by correlations between the ratio of these two principal hydroxycarotenoids in the body and the ratio of pigments deposited in the ornaments (McGraw & Gregory, 2004; García-de Blas, Mateo & Alonso-Alvarez, 2015). Our experimental results also confirm this, and support, to some extent, the hypothesis that carotenoid-based signaling reveals an individual’s capacity to find specific carotenoids in the environment (Hill, 1994; Hill, 2002).

Finally, the fact that astaxanthin and papilioerythrinone were only found in bare parts validates our previous findings (García-de Blas, Mateo & Alonso-Alvarez, 2015) and again supports the idea that biotransformation can take place in situ, at the colored trait, something only explored and described in passerines (McGraw (2004) and McGraw (2009) for eleven species; but see del Val et al. (2009a) and McGraw & Toomey (2010) for two other passerine species). The two recent studies describing a candidate oxygenase for carotenoid biotransformation have detected the enzyme in both the liver and feather follicles of canaries (*Serinus canaria*; Lopes et al., 2016), but only in the bare parts (bill and legs) of zebra finches (Mundy et al., 2016). These differences between only two passerine species would suggest a large diversity in evolutionary constraints and strategies among species.

### Dietary hydroxycarotenoids contributing to color

Lutein and zeaxanthin supplementation attenuated the color decline observed throughout the breeding season in red-legged partridges (Alonso-Alvarez et al., 2008). Consistently with the highest rate of astaxanthin deposition in the ornaments, the ZeaLut treatment produced the reddest birds at the end of the study. We must note that statistical analyses testing the CAR effect only (Table 2) did not include data from birds treated with diquat at the last sampling event, which reduced the sample size by half. When color was tested by controlling the diquat effect, differences between the ZeaLut and LutZea group arose (Tables 3 and 4; Fig. 5). The fact that ZeaLut birds were the reddest suggests that individuals could try to obtain the highest zeaxanthin amounts in the diet to generate ornaments with the highest astaxanthin levels (also García-de Blas, Mateo & Alonso-Alvarez, 2015). This scenario may support the involvement of an allocation trade-off between signaling and self-maintenance functions (Møller et al., 2000) based on a hypothetically scarce resource (i.e. zeaxanthin). On the other hand, the presence of papilioerythrinone in the same ornaments is probably due to the abundance of lutein in the diet and the contribution of papilioerythrinone to color (García-de Blas et al., 2013; García-de Blas et al., 2014). However, astaxanthin is the most conjugated carotenoid, and hence, the reddest (and most abundant) pigment in red-legged partridge ornaments. Nonetheless, it has been shown that variability in papilioerythrinone levels in the red head traits can also contribute to explaining color variation, at least in a correlational sample of these birds (i.e. García-de Blas et al., 2013).

### Oxidative stress and carotenoids

Results support that diquat indeed increased oxidative stress in our birds, although the challenge was apparently mild because no effect on body mass or egg production was detected. Diquat generates superoxide and hydrogen peroxide radicals and has been previously used in the same dose and species, reporting effects on blood antioxidant machinery and lipid peroxidation (Galvan & Alonso-Alvarez, 2009; Alonso-Alvarez & Galván, 2011). In the present study, partridges treated with diquat showed weaker erythrocyte resistance to hemolysis when blood was exposed to another free radical source (AAPH). This measure has been associated with long-term (months or years) survival in other bird species (Alonso-Alvarez et al., 2006; Bize et al., 2014). Moreover, diquat-treated females, but not males, showed higher levels of oxidative damage in plasma lipids. Independently of diquat treatment, females allocated lower carotenoid and tocopherol (i.e. antioxidants) amounts to ornaments than males (sex factor at *P* < 0.05 in most models), suggesting a higher investment in other reproductive traits (e.g. egg yolk). Female birds could be more sensitive to oxidative damage during reproduction due to the costs associated with antioxidant allocation to eggs (e.g. Williams, 2005). Accordingly, female red-legged partridges producing eggs with higher hatching success (probably linked to antioxidant content; McGraw, Adkins-Regan & Parker, 2005) endured higher lipid peroxidation in erythrocytes (i.e. Alonso-Alvarez et al., 2010). Similarly, diquat-treated females, but not males, showed higher lipid peroxidation in the liver than controls, but only among birds that did not receive carotenoid supplements. In fact, LutZea and Ast females treated with diquat even showed a decline in liver MDA values compared to controls of the same group (Fig. 10). This may support the antioxidant role of xanthophylls involved in coloration, at least for females. This role has been questioned repeatedly, at least for avian species (Hartley & Kennedy, 2004; Costantini & Møller, 2008; Isaksson & Andersson, 2008; but see Simons, Cohen & Verhulst, 2012).

Finally, results from circulating hydrosoluble antioxidants (PLAOX) were less consistent, showing declines in response to diquat in some carotenoid groups only, and depending on the sex (Fig. 10). Moreover, independently of diquat effects, higher PLAOX levels in Ast and LutZea birds of both sexes compared to controls were found (Fig. 3). In contrast, PLAOX did not increase in ZeaLut partridges. The antioxidant power of each pigment is linked to the number of conjugated double bonds: 13, 11 and 10 for astaxanthin, zeaxanthin, and lutein, respectively (Britton, 1995; Britton, Liaaen-Jensen & Pfander, 2009; Martínez et al., 2008). Therefore, an increase in PLAOX among ZeaLut birds was predictable. Nonetheless, we must consider that PLAOX mostly assesses the presence of hydro-, but not lipid-soluble antioxidants (Miller et al., 1996; Cohen, Klasing & Ricklefs, 2007). Thus, a higher PLAOX may also be due to a compensatory mobilization of other antioxidants (e.g. vitamin C) to fight off a challenge of some type (Costantini, Metcalfe & Monaghan, 2010). This view particularly agrees with the highest PLAOX values in Ast birds. These animals did not show astaxanthin in plasma and even experienced lower plasma lutein, zeaxanthin and tocopherol levels than controls (above). Similarly, Ast birds did not show astaxanthin in the liver, but accumulated large amounts of vitamin A in this organ, perhaps to protect the liver from some toxic insult (García-de Blas, Mateo & Alonso-Alvarez, 2015). Anyway, we found only one study supporting this toxic effect, in which rats fed with astaxanthin endured an impairment of the liver enzymes involved in detoxification (Ohno et al., 2011). In summary, if PLAOX did not exclusively reveal the antioxidant capacity of circulating carotenoids, the lack of higher PLAOX values in ZeaLut birds could merely be due to other (hydrosoluble) antioxidants being not mobilized. Here the conclusion is that the antioxidant role of carotenoid cannot easily be addressed by PLAOX measures only.

### Oxidative stress and carotenoid biotransformation

Although the proximate cost of ketocarotenoid-based signaling in red-legged birds may, at least partially, involve increased foraging effort to obtain large zeaxanthin amounts (above), the requirement of biotransformation to produce red traits provides another substrate for natural selection. Birds exposed to diquat generated redder bills, which contradicts the constraining impact of oxidative stress on health (e.g. Monaghan, Metcalfe & Torres, 2009; Dowling & Simmons, 2009; Costantini, 2014). The results may, instead, support some response (perhaps hormetic; e.g. Costantini, Metcalfe & Monaghan, 2010) against a mild stressor, at least in terms of color expression, although the exact mechanism can only be deducted (see below).

We must anyway mention that, in contrast to our results, red-legged partridges exposed to the same diquat dose and duration in another experiment, but during the first weeks of life, produced paler red colors in adulthood (Alonso-Alvarez & Galván, 2011). We must nonetheless consider that adverse conditions during early periods of life are particularly damaging (Metcalfe & Monaghan, 2001). Young individuals may not have fully developed antioxidant machinery (Metcalfe & Alonso-Alvarez, 2010) to properly manage such an oxidative challenge. Here, pigment levels in partridge ornaments support the color findings. Carotenoid concentrations increased under diquat exposure. Interestingly, the increase in these tissues was detected for astaxanthin, but not papilioerythrinone.

We can provide two alternative or complementary proximate mechanisms to explain these findings. First, we may suggest that a large availability of superoxide and hydrogen peroxide (free radicals) derived from diquat redox cycling (Koch & Hill, in press) could favor those conditions required for oxygen addition to hydroxycarotenoids by the enzyme (i.e. more than dehydrogenation), and hence, a higher astaxanthin production. We must here remember that astaxanthin production from its substrate requires two oxygenation reactions, whereas papilioerythrinone would require one oxygenation but also a dehydrogenation (McGraw, 2006; LaFountain, Frank & Prum, 2013; García-de Blas et al., 2014). We must also consider that the hypothesized oxygenase (above) should require oxygen, as well as Fe^2+^ cation and nicotinamide adenine dinucleotide phosphate (NADPH; a reducing agent; Fraser, Miura & Misawa, 1997; Schoefs et al., 2001). The second possibility would be that superoxide and hydrogen peroxide levels increased by diquat could act as redox signals (e.g. Hurd & Murphy, 2009) promoting oxygenase (but not dehydrogenase) transcription as a defensive or hormetic mechanism that would ultimately lead to carotenoid biotransformation. The two recent and simultaneously published articles describing the candidate oxygenase for converting yellow to red carotenoids in birds (Lopes et al., 2016; Mundy et al., 2016) show that the enzyme (i.e CYP2J19) is part of the well-known P450 cytochrome, which is involved in many detoxification reactions. Moreover, diquat has been shown to stimulate the transcription of similar oxygenase enzymes (i.e. heme-oxygenases) via redox signaling (i.e. via the Nuclear factor (erythroid-derived 2)-like 2 (Nrf2); e.g. Black et al., 2008; Sun et al., 2011; Wilmes et al., 2011). We may here argue that high superoxide or hydrogen peroxide radicals produced by natural processes (e.g. exercise, flying effort; Costantini, Mirzai & Metcalfe, 2012; Jenni-Eiermann et al., 2014) could activate a similar redox mechanism favoring oxygenase activity, which could explain why wild partridges are redder compared to captive birds whose flying capacity is restrained (García-de Blas, Mateo & Alonso-Alvarez, 2015).

Biotransformation due to oxidative stress, however, seems to be higher among birds with the highest availability of the main ketocarotenoid precursor; that is, ZeaLut birds (see in the eye ring; though *P* = 0.057; Fig. 6). This again supports the importance of acquiring enough quantity of specific carotenoids with the diet in a sexual signaling context (i.e. Hill, 1994; but see Hill, 2011). Nevertheless, the clearest effect was found in diquat-treated birds that did not receive any carotenoid supplementation (Fig. 6). The effect in these two CAR groups would agree with bill color findings (Fig. S2), although the interaction was non-significant. The diquat effect on non-supplemented birds could be due to better zeaxanthin availability in the blood (Fig. 8) and liver (Fig. 9) in this group. Higher circulating levels of zeaxanthin could be a consequence of an active mobilization from stores (liver) and/or better intestinal absorption, both for combating oxidative stress (e.g. Alonso-Alvarez et al., 2008; McClean et al., 2011; but see Isaksson & Andersson, 2008). Recent works suggest that xanthophyll absorption in the intestinal mucosa can be actively regulated by specific protein scavenger receptors such as the class B member 1 (SR-B1; Hill & Johnson, 2012; Sato et al., 2012). How diquat may have favored such receptors can only be speculated, but we could again consider its potential influence on different redox signaling pathways (above; also e.g. Cristóvão et al., 2009; Koch & Hill, in press).

In any event, in order to test whether higher astaxanthin levels in ornaments are due to higher zeaxanthin availability (mobilization) in the body (i.e., not to higher biotransformation rates), we also added plasma or liver zeaxanthin levels as covariates in models testing bill and eye ring astaxanthin concentrations. As expected, a positive link between ornament astaxanthin and plasma zeaxanthin values was observed (also García-de Blas, Mateo & Alonso-Alvarez, 2015), but this did not change the CAR × diquat interaction or post hoc tests (always *P* < 0.05). Moreover, diquat did not increase zeaxanthin values in internal tissues in the other group showing increased astaxanthin deposition in ornaments (ZeaLut; Fig. 8).

Other results may still support the availability of carotenoid precursors as a key factor favoring biotransformation. Diquat decreased tocopherol values in eye rings and bills among birds that did not receive supplemented carotenoids in food (Fig. 7). When tocopherol levels in these bare parts are not statistically controlled for as a covariate, differences in astaxanthin levels among the same control birds (Fig. 6) disappear (both traits: *P* > 0.60), but in the eye rings of ZeaLut birds they become significant (*P* = 0.036). In other words, ZeaLut birds showed the highest astaxanthin levels in eye rings when exposed to diquat. This suggests that biotransformation can be even more stimulated by oxidative stress when the level of carotenoid precursors in the diet surpasses some threshold. When this is not the case, color is not impaired but tocopherol levels are probably consumed to control the challenge.

In summary, the overall results suggest that specific carotenoid precursors must be sufficiently available and that oxidative status must be well-adjusted in order to produce the most pigmented red ornaments. In agreement with this, redder integuments have also been observed in red-legged partridges exposed to other chemicals (i.e. pesticides and heavy metals) that induce oxidative stress (Lopez-Antia et al., 2015a; Lopez-Antia et al., 2015b; Vallverdú-Coll et al., 2015) or in zebra finches enduring experimentally reduced antioxidant (glutathione) levels (Romero-Haro & Alonso-Alvarez, 2015). The findings support the view that oxidative stress is not only a constraint for the expression of optimal phenotypes, but that mild levels are involved in many functions (Jones, 2006; Metcalfe & Alonso-Alvarez, 2010; Isaksson, Sheldon & Uller, 2011). Furthermore, the study supports claims from Hill & Johnson (2012) and Johnson & Hill (2013) hypothesizing that carotenoid-based traits could be signaling an individual’s efficiency to manage oxidative stress. The results also validate the Völker’s (1957) ideas suggesting that a good oxidative metabolism is necessary to biotransform carotenoids used in red coloration, which could explain why birds whose flying capacity was restrained by captivity became paler. However, in contrast to the works of Hill & Johnson (i.e., Hill & Johnson, 2012; Johnson & Hill, 2013), our experiment also suggests the parallel involvement of a resource allocation trade-off because the body levels of substrate carotenoids influenced coloration and even the impact of oxidative stress on biotransformation. In eye rings, under oxidative stress exposure, birds receiving the highest zeaxanthin levels in the diet were also those producing the highest amounts of the main ketocarotenoid (astaxanthin). Finally, we cannot conclude this discussion without applying a life-history perspective. High levels of sexual signaling under high oxidative stress could constitute a sort of terminal investment, with individuals increasing their chances of reproducing when their perception of future survival becomes negative (Velando, Drummond & Torres, 2006; Romero-Haro & Alonso-Alvarez, 2015).

## Supplemental Information

10.7717/peerj.2237/supp-1 Supplemental Information 1 Supplementary material.

10.7717/peerj.2237/supp-2 Supplemental Information 2 Dataset from the diquat experiment. Description of variables inserted as comments on variable names.

10.7717/peerj.2237/supp-3 Supplemental Information 3 Dataset including repeated measures for the carotenoid-supplement experiment. The dataset excludes birds receiving diquat (see Methods). Description of variables inserted as comments on variable names.

## Additional Information and Declarations

### Data Deposition

The following information was supplied regarding data availability: The raw data has been supplied as Supplemental Dataset Files.
