# Supplementary material for: Specific carotenoid pigments in the diet and a bit of oxidative stress in the recipe for producing red carotenoid-based signals
Source: PeerJ. 2016 Sep 1;4:e2237. doi: 10.7717/peerj.2237 (PMC5012267; doi:10.7717/peerj.2237)
Supplement: Supplemental Information 1 [file peerj-04-2237-s001.pdf]

Supplementary material

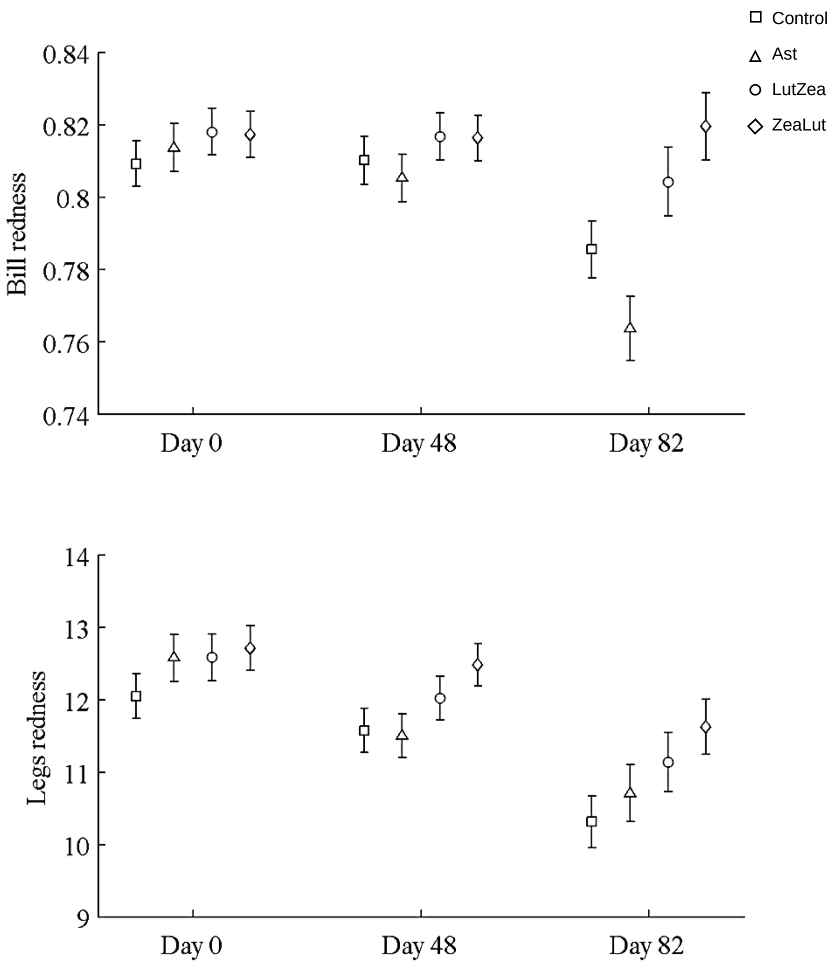

Figure 1SM: *Changes in ornament coloration during the experiment depending on the carotenoid treatment. Least squared means ± se were obtained from the models (see Methods and table 1).*

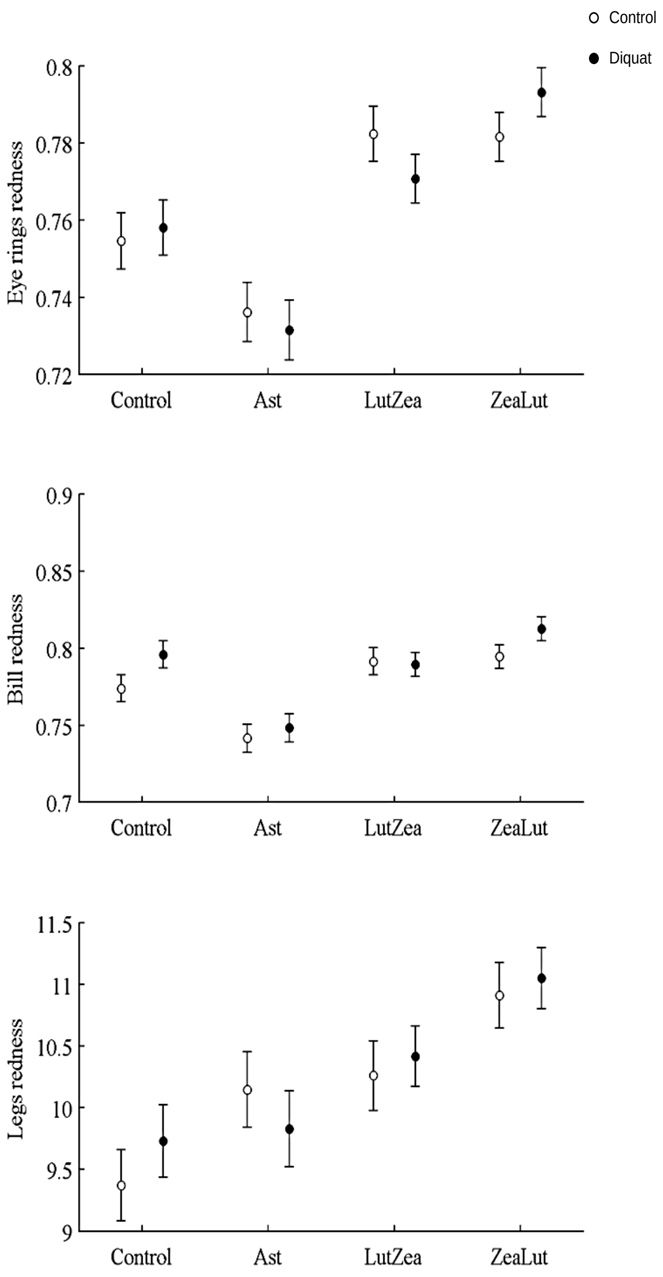

Figure 2SM*: Changes in ornament coloration after diquat exposure depending on the carotenoid treatment. Least squared means ± se from the models (see Methods and table 3).*

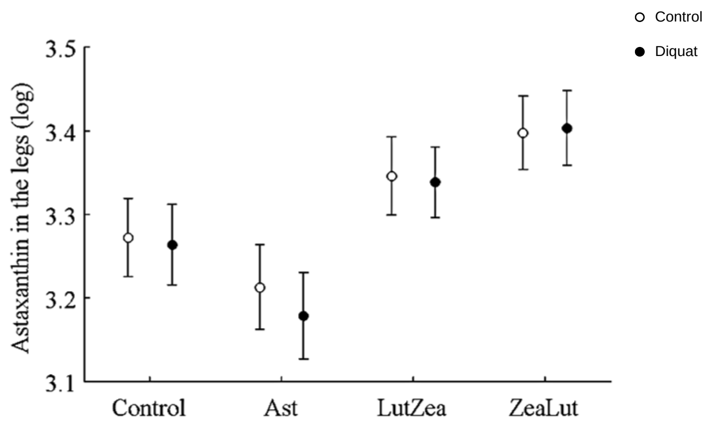

***Figure 3SM**: Levels of astaxanthin in the legs after diquat exposure depending on the carotenoid treatment. Least squared means ± se from the models (see Methods and table 3).*

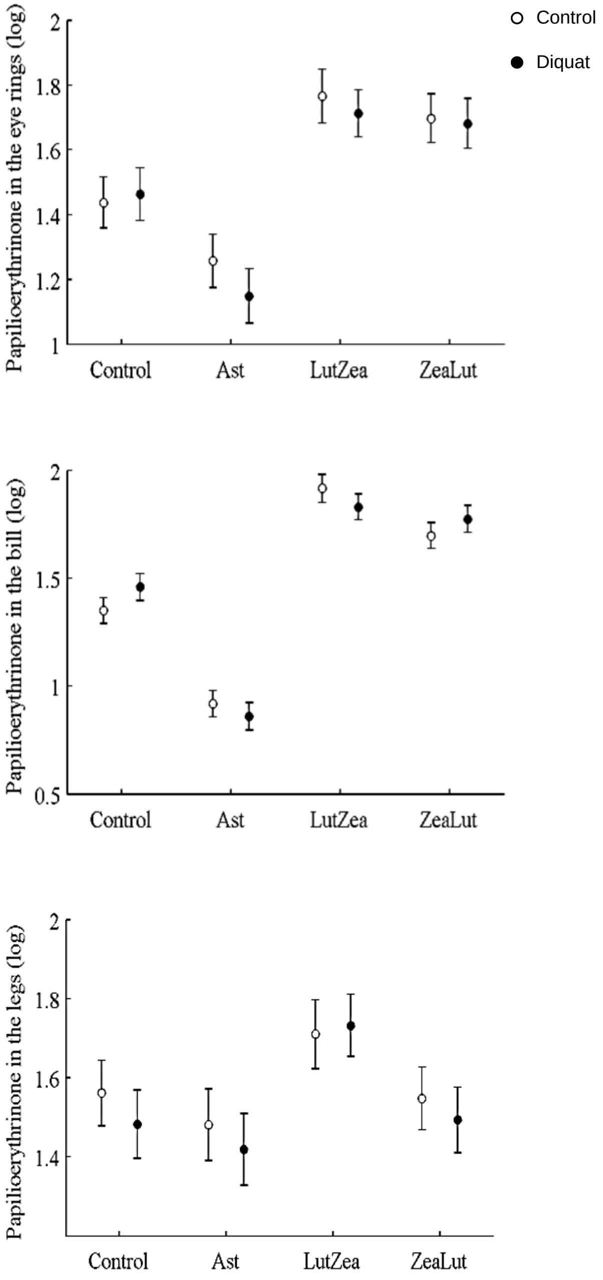

***Figure 4SM**: Levels of papilioerythrinone in the three ornaments after diquat exposure depending on the carotenoid treatment. Least squared means ± se from the models (see Methods and table 3).*

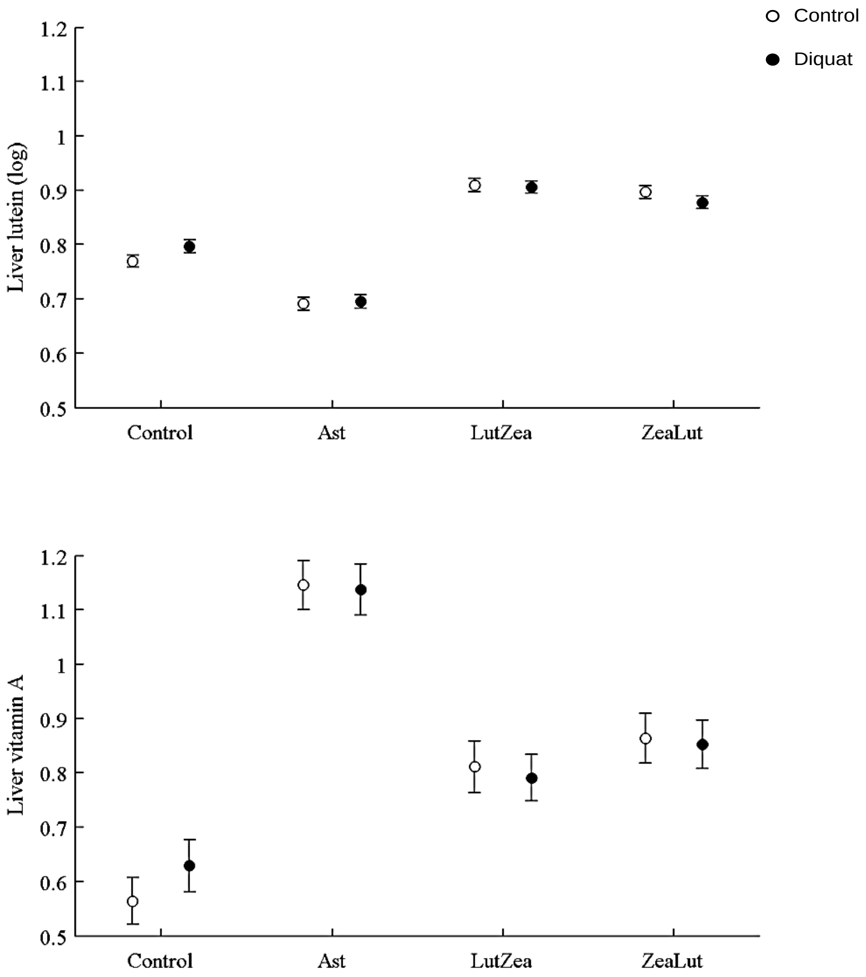

***Figure 5SM**: Levels of lutein and total vitamin A (free and esterified retinol) in liver after diquat exposure depending on the carotenoid treatment. Least squared means ± se from the models (Methods and table 3).*

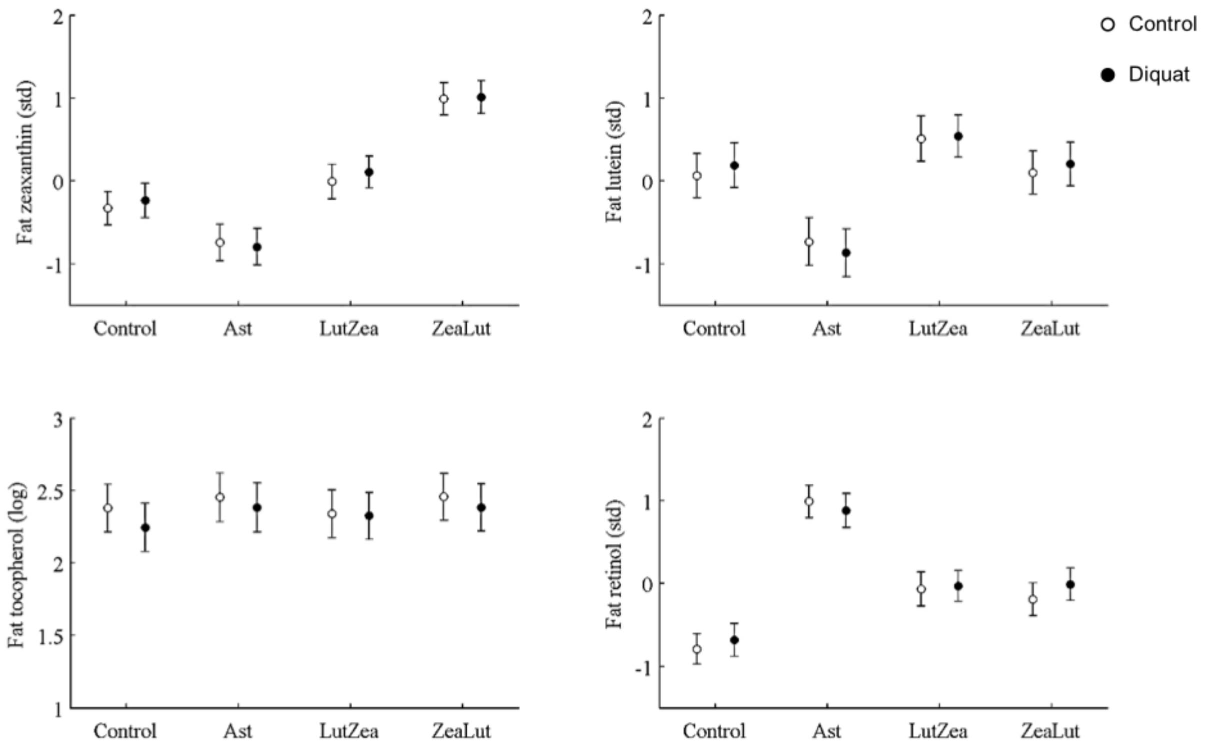

Figure 6SM: *Levels of carotenoids and vitamins in subcutaneous fat after diquat exposure depending on the carotenoid treatment. Least squared means ± se from the models (see Methods and table 3).*

***Table 1SM**: Raw data for carotenoids and vitamins (nmol/ml) in plasma throughout the study. The effects were provided by the models (see table 2). TxC: Time x carotenoid interaction; ns: No significant differences (p> 0.10). Sample size: time 0: N= 173; time 48: N= 173; and time 82: N= 86 (in time 82, diquat birds were discarded, see methods).*

	Time	***Control*** **(mean ± sd)**	***Ast*** **(mean ± sd)**	***LutZea*** **(mean ± sd)**	***ZeaLut*** **(mean ± sd)**	***Effect***
	0	22.57 ± 5.45	21.52 ± 5.54	20.34 ± 6.42	20.16 ± 6.38	
Lutein	48	10.68 ± 2.87	6.56 ± 1.48	16.09 ± 6.10	12.65 ± 3.98	TxC
	82	9.48 ± 1.69	6.52 ± 0.75	16.86 ± 3.55	13.74 ± 2.45	
	0	10.96 ± 2.32	10.64 ± 1.74	10.33 ± 2.46	10.06 ± 2.30	
Zeaxanthin	48	7.64 ± 1.99	6.25 ± 1.58	13.37 ± 5.13	24.37 ± 9.20	TxC
	82	6.74 ± 1.07	6.59 ± 0.93	13.56 ± 3.28	25.88 ± 5.07	
	0	22.35 ± 9.67	22.73 ± 6.66	21.18 ± 8.15	21.37 ± 9.49	
Tocopherol	48	9.08 ± 3.29	9.30 ± 3.24	10.50 ± 4.65	11.83 ± 4.70	TxC
	82	11.02 ± 2.68	11.02 ± 2.56	12.60 ± 3.63	14.91 ± 3.99	
	0	23.89 ± 3.22	24.05 ± 4.21	24.08 ± 4.45	22.92 ± 3.57	
Retinol	48	22.11 ± 3.42	23.32 ± 3.62	23.78 ± 4.56	23.80 ± 4.49	ns
	82	25.74 ± 4.71	27.59 ± 4.44	27.01 ± 3.94	27.42 ± 4.38	

Table 2SM: *Raw data for carotenoids and vitamins (nmol/g) in ornaments after the diquat exposure. The effects were provided by the models (see tables 3 and 4). C: Group of carotenoid supplement; DxC: Diquat x carotenoid interaction; D: Diquat treatment; S: Sex*

			Control **(mean ± sd)**	***Ast*** **(mean ± sd)**	***LutZea*** **(mean ± sd)**	***ZeaLut*** **(mean ± sd)**	***Effect***
**Eye rings**	*Astaxanthin*	Control	66.68 ± 38.04	69.64 ± 28.80	113.57 ± 63.17	168.45 ± 74.50	DxC
		Diquat	61.52 ± 32.93	44.31 ± 20.24	103.46 ± 53.79	225.62 ± 141.05	
	Papilioerythrinone	Control	7.63 ± 4.31	3.73 ± 2.42	16.59 ± 8.45	12.91 ± 5.26	C, S
		Diquat	6.30 ± 3.77	2.69 ± 2.01	16.65 ± 10.20	16.60 ± 10.55	
	Tocopherol	Control	63.91 ± 69.12	62.12 ± 23.49	45.10 ± 18.32	54.61 ± 20.44	DxC
		Diquat	39.21 ± 15.31	50.25 ± 16.65	45.48 ± 14.22	58.56 ± 19.50	
**Bill**	*Astaxanthin*	Control	156.80 ± 76.32	100.93 ± 36.08	373.51 ± 148.63	444.05 ± 287.12	DxC
		Diquat	148.61 ± 75.94	102.73 ± 50.98	224.45 ± 106.71	517.99 ± 276.06	
	Papilioerythrinone	Control	15.10 ± 9.85	6.02 ± 3.87	52.31 ± 31.08	26.06 ± 14.90	C, S
		Diquat	13.08 ± 6.36	4.72 ± 2.84	35.03 ± 24.01	32.67 ± 15.41	
	Tocopherol	Control	84.42 ± 20.90	88.06 ± 23.40	91.98 ± 26.96	82.22 ± 26.64	DxC
		Diquat	65.46 ± 25.05	87.06 ± 28.84	71.20 ± 30.59	89.90 ± 28.68	
**Legs**	*Astaxanthin*	Control	49.07 ± 41.32	41.68 ± 25.82	71.45 ± 22.61	106.47 ± 70.17	C, S
		Diquat	36.64 ± 24.77	40.88 ± 49.75	60.27 ± 41.63	107.89 ± 70.37	
	Papilioerythrinone	Control	6.07 ± 3.75	4.41 ± 2.77	10.25 ± 2.55	8.29 ± 4.32	C, S
		Diquat	5.12 ± 2.47	3.51 ± 4.48	9.71 ± 5.74	8.97 ± 7.54	
	Tocopherol	Control	53.78 ± 15.96	54.68 ± 11.53	46.26 ± 12.59	54.69 ± 16.50	C, S
		Diquat	47.31 ± 12.84	48.53 ± 16.14	48.85 ± 16.19	55.73 ± 19.75	

Table 3SM: *Raw data for carotenoids and vitamins in plasma (nmol/mL), liver and fat (nmol/g) after the diquat exposure. The effects were provided by the models (see tables 3 and 4). C: Group of carotenoid supplement; DxC: Diquat x carotenoid interaction; S: Sex; ns: No significant differences.*

			Control **(mean ± sd)**	***Ast*** **(mean ± sd)**	***LutZea*** **(mean ± sd)**	***ZeaLut*** **(mean ± sd)**	***Effect***
Plasma	*Lutein*	Control	9.48 ± 1.69	6.52 ± 0.75	16.86 ± 3.55	13.74 ± 2.45	C
		Diquat	9.45 ± 1.66	6.55 ± 0.77	15.03 ± 4.23	12.80 ± 2.67	
	Zeaxanthin	Control	6.74 ± 1.07	6.59 ± 0.93	13.56 ± 3.28	25.88 ± 5.07	C
		Diquat	6.79 ± 1.09	6.82 ± 1.31	12.66 ± 4.46	24.52 ± 6.12	
	Retinol	Control	25.74 ± 4.71	27.59 ± 4.44	27.01 ± 3.94	27.42 ± 4.38	ns
		Diquat	24.71 ± 4.41	26.07 ± 3.94	26.86 ± 3.02	27.45 ± 4.19	
	Tocopherol	Control	11.02 ± 2.68	11.02 ± 2.56	12.60 ± 3.63	14.91 ± 3.99	C, D
		Diquat	9.46 ± 3.65	11.14 ± 4.04	11.97 ± 3.95	13.71 ± 3.89	
Liver	*Lutein*	Control	5.75 ± 0.49	4.86 ± 0.36	8.31 ± 1.54	8.82 ± 1.99	C
		Diquat	5.70 ± 0.63	4.83 ± 0.39	8.14 ± 1.80	8.20 ± 2.22	
	Zeaxanthin	Control	4.16 ± 0.34	4.11 ± 0.48	6.52 ± 1.23	14.21 ± 3.80	DxC
		Diquat	4.11 ± 0.38	4.12 ± 0.53	6.29 ± 1.35	12.84 ± 4.45	
	Total vitamin A	Control	389.28 ± 245.49	1392.40 ± 490.99	679.07 ± 237.45	814.33 ± 325.32	C, S
		Diquat	449.32 ± 297.65	1374.66 ± 682.11	618.66 ± 269.50	765.81 ± 399.72	
	Tocopherol	Control	6.02 ± 1.69	6.67 ± 2.67	6.75 ± 2.66	9.03 ± 2.52	DxC
		Diquat	4.71 ± 1.05	6.45 ± 2.68	6.65 ± 2.63	8.10 ± 3.76	
Fat	*Lutein*	Control	17.74 ± 12.92	21.78 ± 13.77	25.31 ± 12.53	18.54 ± 13.63	C
		Diquat	18.19 ± 11.16	19.95 ± 9.77	20.79 ± 16.24	21.46 ± 13.70	
	Zeaxanthin	Control	11.56 ± 8.25	15.37 ± 9.46	16.30 ± 7.63	16.07 ± 11.12	C
		Diquat	11.98 ± 7.17	14.43 ± 6.98	13.89 ± 10.51	18.75 ± 11.71	
	Retinol	Control	87.09 ± 64.70	196.73 ± 108.90	149.17 ± 79.36	108.26 ± 86.98	C
		Diquat	98.36 ± 65.76	179.52 ± 99.98	116.00 ± 99.21	131.30 ± 90.95	
	Tocopherol	Control	381.21 ± 392.46	489.32 ± 599.44	328.65 ± 297.64	319.61 ± 260.11	ns
		Diquat	267.97 ± 283.51	396.92 ± 341.49	318.61 ± 378.15	373.70 ± 384.49	

Table 4SM: *Raw data for oxidative stress biomarkers in blood and other tissues at the end of the study after diquat treatment. The effects were provided by the models (see tables 3 and 4). C: Group of carotenoid supplement; D: Diquat treatment; DxCxS; Diquat x carotenoid x sex interaction; DxS: Diquat x Sex interaction.*

	Sex		***Control (mean ± sd)***			***Ast (mean ± sd)***			***LutZea (mean ± sd)***			***ZeaLut (mean ± sd)***			***Effect***
Resistance to oxidative stress in erythrocytes (min)		Control	101.38	±	10.4	106.25	±	11.89	103.67	±	12.093	103.13	±	11.64	D
		Diquat	100.2	±	11.7	97.211	±	10.04	100	±	8.56	99.3	±	9.739	
Plasma TRG-MDA-corrected (µM)	Male	Control	3.387	±	0.687	2.954	±	0.573	3.311	±	0.490	3.458	±	0.965	DxS
		Diquat	3.879	±	0.521	3.840	±	0.475	4.057	±	0.503	4.102	±	0.824	
	Female	Control	3.660	±	2.374	3.012	±	0.514	3.114	±	0.543	3.408	±	0.615	
		Diquat	4.619	±	1.894	4.330	±	1.550	4.159	±	1.337	4.517	±	2.523	
UA-ALB-corrected PLAOX (mmol/L)	Male	Control	0.008	±	0.089	0.079	±	0.125	-0.038	±	0.103	-0.026	±	0.102	DxCxS
		Diquat	0.020	±	0.072	0.104	±	0.114	0.007	±	0.080	-0.036	±	0.097	
	Female	Control	0.001	±	0.160	-0.008	±	0.091	-0.047	±	0.078	-0.011	±	0.176	
		Diquat	-0.004	±	0.076	0.060	±	0.224	-0.043	±	0.042	-0.043	±	0.067	
Liver MDA (µmol/g)	Male	Control	0.026	±	0.007	0.023	±	0.006	0.026	±	0.005	0.024	±	0.005	DxCxS
		Diquat	0.023	±	0.006	0.023	±	0.007	0.025	±	0.007	0.026	±	0.007	
	Female	Control	0.025	±	0.007	0.034	±	0.010	0.033	±	0.014	0.028	±	0.004	
		Diquat	0.032	±	0.006	0.030	±	0.012	0.024	±	0.007	0.028	±	0.010	
Heart MDA (µmol/g)		Control	0.025	±	0.005	0.023	±	0.007	0.027	±	0.01	0.026	±	0.011	C
		Diquat	0.024	±	0.005	0.027	±	0.005	0.023	±	0.006	0.024	±	0.005	
